# Pangenomic Approach for the Identification and Functional Characterization of Active GASA Antimicrobial Genes in Citrus Rootstocks for Resistance Breeding Against Bacterial Pathogens

**DOI:** 10.3390/plants15030425

**Published:** 2026-01-30

**Authors:** Florencia Nicole Bekier, Mariana Conte, Rodrigo Machado, Lourdes Pereyra Ghidela, Natalia Inés Almasia, Vanesa Nahirñak, Nadia Frías, Paula del Carmen Fernández, Cecilia Vazquez Rovere, Horacio Esteban Hopp, Gabriela Conti

**Affiliations:** 1 Instituto de Agrobiotecnología y Biología Molecular (IABIMO), Unidad Ejecutora de Doble Dependencia, Consejo Nacional de investigaciones Científicas y Técnicas, Instituto Nacional de Tecnología Agropecuaria (UEDDCONICET-INTA) and Instituto de Biotecnología, Centro de Investigaciones de Ciencias Veterinarias y Agronómicas, Instituto Nacional de Tecnología Agropecuaria (INTA), Hurlingham B1686, Argentina; conte.mariana@inta.gob.ar (M.C.); rodrigomachadobirollo@gmail.com (R.M.); pereyra.lourdes@inta.gob.ar (L.P.G.); almasia.natalia@inta.gob.ar (N.I.A.); nahirnak.vanesa@inta.gob.ar (V.N.); nadia.frias.91@gmail.com (N.F.); fernandez.pc@inta.gob.ar (P.d.C.F.); vazquez.cecilia@inta.gob.ar (C.V.R.); hopp.esteban@inta.gob.ar (H.E.H.); 2 Facultad de Ciencias Exactas y Naturales, Universidad de Buenos Aires, Intendente Güiraldes 2160, Buenos Aires C1428, Argentina; 3 Facultad de Agronomía, Cátedra de Genética, Universidad de Buenos Aires, Av. San Martín 4453, Buenos Aires C1417, Argentina

**Keywords:** SNAKIN/GASA, *Pseudomonas syringae*, *Xanthomonas citri*, *Poncirus trifoliata*, antimicrobial protein, *Citrus* germplasm, *Nicotiana benthamiana*, huanglongbing, hypersensitive reaction, agroinfiltration

## Abstract

The SNAKIN/GASA family comprises antimicrobial peptides with proven activity against bacteria and fungi, making them promising candidates for improving disease resistance in citrus rootstocks. In sixty-seven new GASA variants from a citrus germplasm collection, the presence of the characteristic 12-cysteine domain was confirmed and were classified into three subfamilies. The absolute expression levels of ten representative genes were analyzed in floral tissues, young leaves, and mature leaves from five citrus accessions with contrasting susceptibility to *Xanthomonas citri*. Expression profiling revealed tissue-specific patterns, with higher transcript abundance in juvenile and floral tissues of tolerant accessions. Meta-analysis of HLB-related RNA-seq datasets revealed the upregulation of specific GASA genes. Three genes from *Poncirus trifoliata*—*Pt*GASA6, *Pt*GASA8, and *Pt*GASA10—were then selected for functional validation in *Nicotiana benthamiana*. Transient overexpression of *Pt*GASA6 and *Pt*GASA10 significantly reduced disease symptoms caused by *Pseudomonas syringae* and heightened the hypersensitive response to *X. citri*, whereas *Pt*GASA8 showed no detectable effect. Notably, *Pt*GASA6 enhanced the hypersensitive response by 30% more than *Pt*GASA10, while *Pt*GASA10 delayed necrosis by 40% more than *Pt*GASA6, indicating distinct antimicrobial mechanisms. Together, these results identify *Pt*GASA6 and *Pt*GASA10 as strong candidates for breeding and biotechnological strategies aimed at improving broad-spectrum bacterial disease resistance in citrus.

## 1. Introduction

Bacterial diseases such as huanglongbing (HLB) and citrus canker continue to threaten the sustainability of citrus production worldwide, challenging conventional breeding and demanding robust genetic targets for improved resistance for breeding [1,2,3,4,5,6]. To that end, identification of key genetic targets conferring such resistance requires the screening of the wide *Citrus* pangenome diversity stored in germplasm banks. A comprehensive co-expression network analysis was recently generated using RNA-seq data from 17 public datasets offering a molecular framework for understanding HLB pathogenesis and host response [7].

Among these key genetic targets, different antimicrobial peptide (AMP) families have been profiled to explore new pathways for the control of destructive bacterial diseases like HLB [8] to identify and introgress their encoding gene variants from resistant genotypes into the cultivated susceptible varieties.

The SNAKIN/GASA gene family encodes small AMPs with such potential, since several studies have demonstrated their antimicrobial role against pathogenic agents of various origins, including bacteria and fungi [2,9,10,11,12,13]. It represents a well-characterized group of cysteine-rich AMPs with multiple members and proven activity against bacterial and fungal pathogens in various plant species [2,10,11,14,15,16,17,18,19,20,21]. Some authors consider them to be the type of AMPs with the greatest effect against fungal and bacterial pathogens [9]. These peptides exhibit broad-spectrum activity against bacterial and fungal pathogens and are implicated in diverse physiological processes, including growth regulation and stress responses [2,10,11,18,22,23].

Recent genomic surveys have revealed a large repertoire of GASA genes in citrus, with at least 18 annotated members and additional variants such as GASA9-like [24]. There are examples showing that the introduction of coding genes for antimicrobial peptides into citrus is a suitable alternative for the control of pathogenic microorganisms [2,16,25]. Yet, functional characterization in citrus remains scarce, and the extent to which these genes contribute to broad-spectrum or specific immunity is unclear. Given the urgent need for durable resistance and the complexity of citrus breeding, identifying and validating endogenous GASA genes with antimicrobial activity could provide a strategic advantage for both conventional and biotechnological improvement. However, their antimicrobial mechanisms of action are not yet understood, in part due to their pleiotropic effects, which severely affect the architectural phenotype in mutant plants [10,18,22,26].

The aim of this work is to identify and characterize new candidate endogenous genes by exploring the pangenomic diversity of defense mechanisms among GASA genes present in *Citrus* rootstock germplasm, like *Poncirus trifoliata.* These findings could contribute to breeding efforts by introgression or genomic editing of new resistance genes or alleles (yet to be discovered) present in germplasm accessions. In potato (*Solanum tuberosum*), members such as Snakin-1 and Snakin-3 have demonstrated strong antimicrobial effects and have been successfully deployed in transgenic strategies to reduce disease symptoms [10,18,20]. However, despite their potential, the mechanisms of action of GASA proteins remain poorly understood, partly due to pleiotropic effects that complicate functional studies.

Conventional breeding for disease resistance in citrus is notoriously difficult because of biological constraints, including long juvenile phases, apomixis, partial sterility, and frequent interspecific hybridization difficulty [4,6].

These limitations have shifted breeding efforts toward rootstock improvement, as tolerant rootstocks can confer resilience to the grafted scion [14,27]. However, different citrus accessions used as rootstocks were shown to confer very contrasting resistance against bacterial diseases. Among these, trifoliate orange (*P. trifoliata*) stands out as a key genetic resource due to its sexual compatibility with *Citrus* species and its documented tolerance to HLB and other diseases [1,3,5,28,29].

In this paper, a pangenomic approach to explore the diversity of GASA genes in citrus rootstock germplasm and to identify candidates with potential antimicrobial roles was performed. The cloning and classification of 67 new GASA genomic variants across eight *Citrus* and related species were reported, their conservation within three major subfamilies was confirmed, and their expression patterns in tissues relevant to defense and development were analyzed. To bridge genomic discovery with functional evidence, three candidates—*Pt*GASA6, *Pt*GASA8, and *Pt*GASA10—were selected from *P. trifoliata*, a rootstock known for its disease tolerance, and their activity was evaluated in a model pathosystem using *Nicotiana benthamiana* challenged with *Xanthomonas citri* (incompatible interaction) and *Pseudomonas syringae* pv. *tabaci* (compatible interaction). These findings provide new insights into the structural and functional diversity of citrus GASA proteins and highlight promising genetic targets for breeding and genome editing strategies aimed at improving bacterial disease resistance.

## 2. Results

### 2.1. Identification and Classification of New and Old Genomic Variants of Citrus GASA Genes

**Figure 1:**
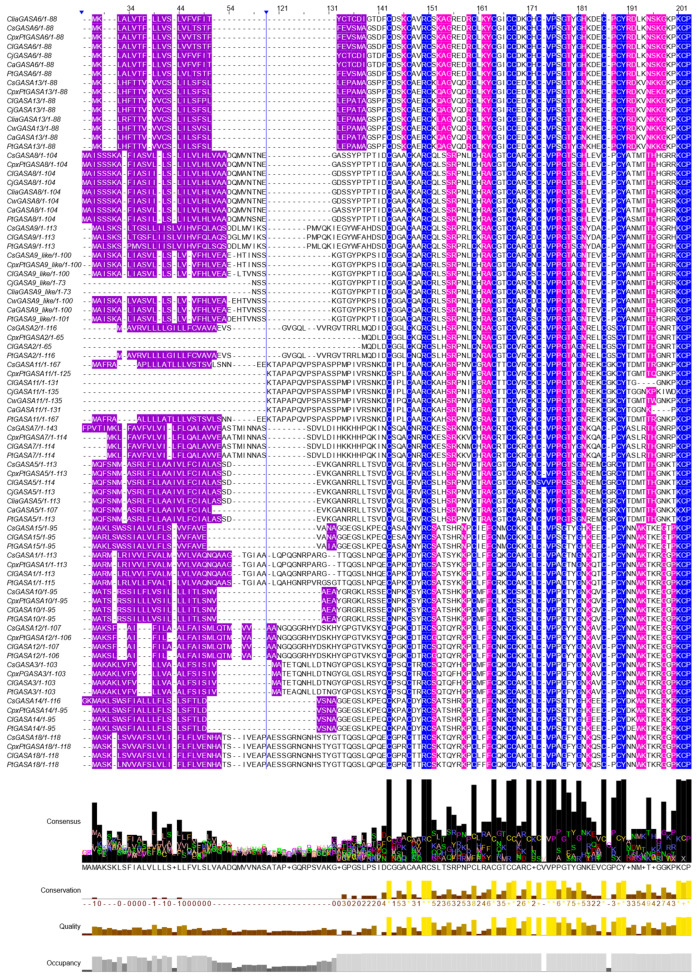
Multiple alignment of the predicted amino acid sequences of citrus GASA protein family. It was performed using Clustal Omega version 1.2 and colored with the Jalview software version 2. Conserved sequences of the SNAKIN/GASA domain are marked in blue and include the conserved characteristic array of 12 cysteines. Residues used to classify each member into a subfamily are shown in magenta, and putative signal peptide-associated sequences, according to Nahirñak et al. [30], are shown in violet. The vertical blue lines indicated with arrows represent variable regions that were hidden for illustrative purposes. Highly conserved amino acids obtained from the ClustalO version 1.2 alignment in Jalview version 2 are shown and include the twelve cysteines, together with the final C-terminal KCP motif.

By using PCR primers targeting conserved SNAKIN/GASA consensus DNA sequences, 67 different native genomic sequence variants present in a core *Citrus* germplasm bank collection were cloned and confirmed by DNA sequencing (Table 1 and Figure 1).

Due to the difficulty of performing genetic crossing experiments and the high genomic diversity of the germplasm accessions (including interspecific hybrids, polyploids, and diploid heterozygotic species) [4,6,14], it is challenging to differentiate polymorphic alleles of a single gene from different gene copies belonging to a related gene family (as in the case of GASA9 and GASA9-like). As expected, the variants that were cloned and sequenced in this work belong to both types (alleles of the same gene or paralog copies of different genes within the family). In Supplementary Figure S1, possible bioinformatic interpretation errors that may occur during gene annotation of new sequences into the existing gene nomenclature are illustrated. These 67 cloned genomic sequences were grouped into 16 different putative genes and designated according to the *Citrus clementina* GASA gene classification established by Wu et al. [24]. Sixteen of them (those present in *Citrus sinensis*, *Citrus limon* and *P. trifoliata* accessions) showed DNA and predicted protein sequences essentially identical to those annotated in the latest version of the reference *Citrus* sp. scaffolds [31], with just minor differences in their aminoacidic sequence. For citrumelo (*Citrus paradisi* × *P. trifoliata*), 14 new GASA gene variants were cloned and sequenced. For *Citrus jambhiri* and *Citrus warburgiana*, the GASA genes cloned and sequenced were five for each of them (GASA6, 13, 5, 8, and 9-like; and 13, 11, 5, 8, and 9-like, respectively). For *Citrus limonia* and *Citrus aurantium* were six (GASA6, 13, 11, 5, 8, and 9-like, for both). The list of sequence names, gene IDs, number of exons, number of nucleotides in their coding sequences (CDS), and amino acids in the encoded protein are summarized in Table 1. Likewise, specific conserved domains allowed the subdivision into three subfamilies. Subfamily I showed two members: GASA6 and GASA13 (annotated as peamaclein). Subfamily II was represented by seven members: GASA8 (annotated as Gibberellin regulated protein 11), GASA9 (annotated as Snakin-2 or gibberellin-regulated protein 1-like), GASA9-like (annotated as gibberellin-regulated protein 1), GASA11 (annotated as gibberellin-regulated protein 14), GASA2 (annotated as Snakin-2 or gibberellin-regulated protein 3), GASA7 (annotated as snakin-2 isoform X1), and GASA5 (annotated as snakin-2-like). The subfamily III also showed seven members: GASA15 (annotated as protein GAST1), GASA1 (annotated as protein GAST1-like), GASA10 (annotated as gibberellin-regulated protein 5), GASA12 (annotated as gibberellin-regulated protein 4), GASA3 (annotated as gibberellin-regulated protein 6), GASA14 (annotated as gibberellin-regulated protein 12-like), and GASA18 (annotated as protein GAST1).

### 2.2. Location of GASA Genes in Chromosomes and Synteny Blocks

**Figure 2:**
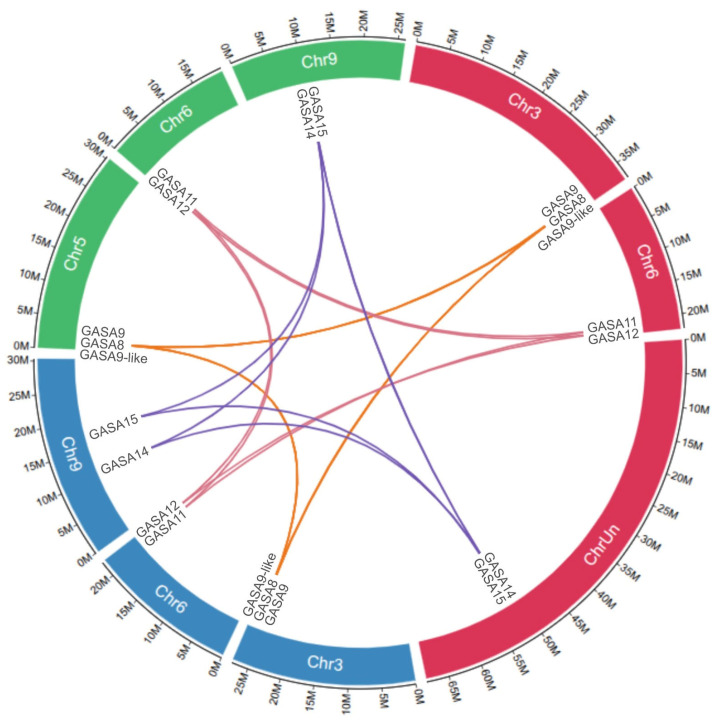
Syntenic analysis of GASA family genes in *Citrus sinensis*, *Citrus limon*, and *Poncirus trifoliata*. Genes displayed on circular bar-blocks indicate the chromosomal position: *C. sinensis* (red), *P.trifoliata* (green), and *C. limon* (blue) chromosomes. Violet, yellow, and red color lines represent duplicated pairs.

These 16 identified GASA genes were mapped into six of the nine chromosomes of *C. sinensis* or into the seven chromosomes of *C. limon* and *P. trifoliata*, according to the known sequence available in databases (see Supplementary Figure S2). The DNA sequences of the first two species are currently used as reference genomes—together with the one of *C. clementina*—because they were the first to be published, thoroughly curated, and their genes are presented within the frame of their completely resolved haplotypes and mapped to their corresponding chromosomes [32,33,34]. Although substantially more DNA sequence information for other *Citrus* species was published later (including *P. trifoliata*), these two specific reference genomes were selected because they represent the most complete and best curated assemblies available. GASA8, 9, and 9-like were mapped to chromosome 3 in the *C. sinensis* and *C. limon* reference genomes, while in *P. trifoliata*, they were mapped to chromosome 5. These three mentioned genes are part of a syntenic region in the chromosomes of *C. limon*, *C. sinensis* and *P. trifoliata*, where they show a common order of homologous genes and conservation of chromosomal sequences. In *C. clementina* only GASA8 and 9 have been annotated to date, but they are also joined in a syntenic block, like in *C. limon*, *C. sinensis*, and *P. trifoliata*. In a similar way, GASA11 and 12 are grouped in a syntenic block in *C. sinensis*, *C. limon*, and *P. trifoliata*, as published in *C. clementina*. In the same way, GASA14 and 15 are grouped in another syntenic block (see Figure 2).

### 2.3. Phylogenetic Analysis of GASA Genes

**Figure 3:**
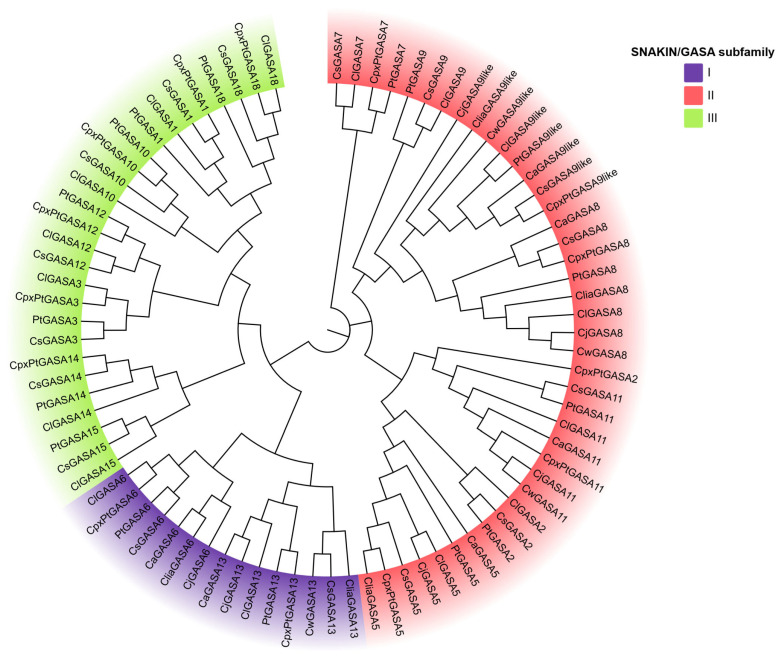
Phylogenetic analysis of citrus GASA proteins. It was generated using MEGA 11 with the Neighbor-Joining method and 1000 bootstrap replicates. A colored version was produced using the online tool ChiPlot tool version 2.6.1. Subfamilies are indicated with colors: subfamily I in violet, subfamily II in magenta, and subfamily III in green. The varieties included in this analysis are: *Cs*, *Citrus sinensis*; *Cp* × *Pt*, Citrumelo (*Citrus* × *paradisi* × *Citrus trifoliata*); *Cl*, *Citrus limon*; *Pt*, *Poncirus trifoliata*; *Cj*, *Citrus jhambiri*; *Clia*, *Citrus limonia*; *Cw*, *Citrus warburgiana*; *Ca*, *Citrus aurantium*.

The phylogenetic relationship among all GASA proteins obtained from eight different *Citrus* species was resolved in the dendrogram shown in Figure 3. As expected, all of them clustered with their orthologs, i.e., the members of each subfamily grouped together. The evolutionary history was inferred using the Neighbor-Joining method [35].

### 2.4. Differential Expression of Selected Citrus GASA Genes in Juvenile, Mature Leaves, and Floral Tissues

**Figure 4:**
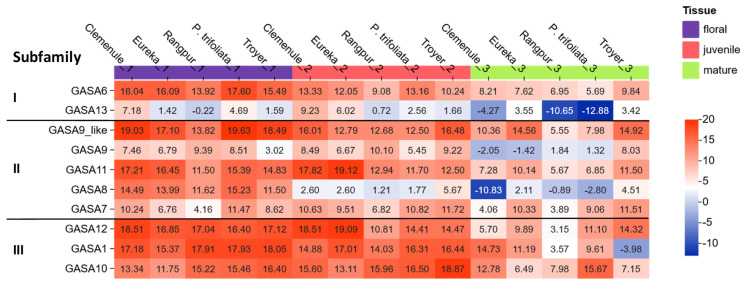
Representative heatmap of gene expression. It was obtained through absolute RT-qPCR from floral tissue (purple), juvenile (magenta), and mature leaf (green) of citrus cultivars *C. reticulata* var ‘Clemenule’, *Citrus limon* var. ‘Eureka’, *Citrus limonia* (Rangpur), *Citrange Troyer*, and *Poncirus trifoliata*. The heatmap, generated using the Chiplot tool, employs a color scale ranging from dark blue, representing minimum expression, to dark red, representing maximum expression. On the left, the analyzed GASA genes are shown along with their corresponding subfamily classification (I, II, or III).

Different *Citrus* accessions were shown to confer very contrasting susceptibility against bacterial diseases [1,3,5]. Wu et al. [24] reported that GASA genes have a detectable basal gene expression in different tissues of a known cultivated variety of *C. clementina*. Based on these results, a similar approach for the *Citrus* species analyzed in this paper was followed, with particular interest in the rootstock accessions used for breeding showing different levels of tolerance to bacterial diseases. To gain insight into the putative involvement of these new GASA candidate genes in broad-spectrum immunity, their expression was measured in juvenile tissues—specifically young leaves and floral tissues, which are expected to exhibit higher expression levels—versus mature leaves from *C. limon* var ‘Eureka’, *C. reticulata* var ‘Clemenule’, *Poncirus trifoliata*, and Citrange Troyer and Rangpur by absolute quantitative RT-PCR (see Figure 4). This technique was used to quantify the number of transcripts corresponding to each of the genes and normalized for comparison of the baseline expression levels. Resulting absolute gene expression levels are represented by the heatmap shown in Figure 4.

In general, heterogeneous results were observed depending on the specific combination of GASA gene, plant genotype, and tissue analyzed. This variability becomes also evident in the next section, where the nucleotide sequence structure of the promoters is analyzed. Thus, heterogeneity could be attributed to the presence of multiple putative cis-regulatory elements in GASA gene promoters, which are potentially influenced by tissue specificity, developmental stage, hormones, light, and other environmental factors. However, taking all the data together, there is a clear tendency for flower tissue and juvenile leaves to show (on average) higher levels of expression than mature leaves, suggesting that both tissue and ontology specificity are the predominant factors influencing expression. Overall, GASA genes with the highest expression levels (shown in dark red) were found in the tissues of interest (young leaf and/or floral tissue) in agreement with previous observations [36]. In contrast, the lowest expression levels were found in mature leaves (dark blue color). Among the genes with the highest accession-specific expression were GASA9-like, 10, 11, and 12. On the contrary, GASA13 showed the lowest expression level in general. When considering GASA genes all together for most accessions, the best expression levels were observed for GASA1, 6, 9-like, 10, 11, and 12. Finally, when levels of expression were considered for each specific genotype, *P. trifoliata*, Citrange Troyer, and Clemenule showed the highest tissue specific expression in flower tissue and young leaves.

At least one representative member from each subfamily branch was selected to continue the functional characterization, regardless of the comparative level of basal expression between subfamilies. In addition, alternative criteria beyond high expression in young leaves or floral tissue of the plant were considered (within each subfamily). For instance, GASA6 (from subfamily I) was selected because it is the closest relative member to Snakin1 from *Solanum tuberosum* (*St*SN1), which is well known as an antimicrobial peptide against multiple fungi and bacteria in different pathosystems [2,10,20,37,38] and showed better expression levels than its alternative in subfamily I (GASA13), which exhibited the lowest expression levels. Likewise, GASA8 and 10 are orthologs of *St*SN2 and *St*SN3, respectively. GASA8 presented a good expression in the floral tissue of *P. trifoliata* (an HLB-tolerant rootstock) and is also the only one that has a decreased expression in Rangpur, a rootstock with greater susceptibility to pathogens [4,5]. GASA10 showed a markedly higher expression in the most resistant rootstock accessions (Citrange Troyer and *P. trifoliata*) and contrasting low levels in two of the most susceptible cultivars (*C. clementina* and *C. limon* var. Eureka).

**Figure 5:**
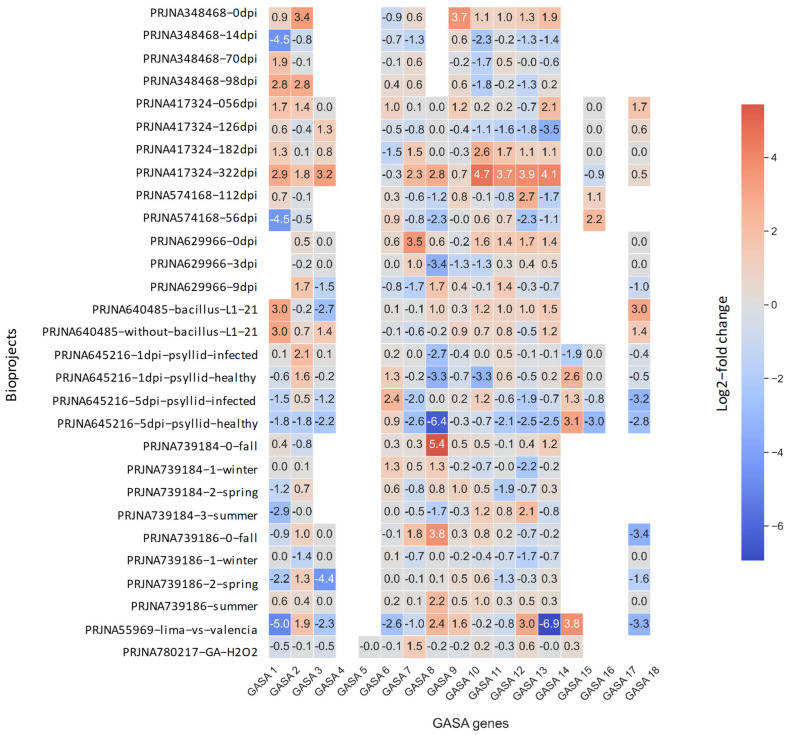
Heatmap showing the log_2_-fold change of 18 citrus GASA genes across public HLB-related RNA-seq BioProjects. Each cell represents the log_2_-fold change of a given GASA gene in a specific contrast. Red shades indicate GASA overexpression (positive log_2_FC), blue shades indicate under-expression (negative log_2_FC), and near-white cells correspond to little or no change. Empty cells reflect cases where the corresponding GASA gene was not detected in that dataset or lacked sufficient read support to generate a reliable log_2_FC estimate. In Supplementary Table S1, there is a description of the experimental approach of each BioProject and their specific references. In Supplementary Figure S2, some specific methodological issues regarding bioinformatic gene annotations performed by individual Bioprojects for GASA16–18 are shown.

Wu et al. [24] also reported that, after experimental infection with *X. citri* in detached leaf explants of *C. clementina*, the expression of certain GASA genes is significatively increased, including *Cc*GASA8 (among other nine genes). The induction was therefore investigated using alternative bacterial pathogens, such as *Candidatus* Liberibacter asiaticus, the causal bacterium associated with HLB disease. Because of its economic importance, there is a good deal of BioProjects deposited in public databanks like DDBJ, EMBL–EBI, and NCBI, as described in Supplementary Table S1. Such differential expression profiling can be associated with a response to HLB disease both, in susceptible *Citrus* cultivar species like *C. sinensis* or in HLB-tolerant species like *C. australasica* [8,39]. In this context, recent publications report the upregulation of AMP genes after infection of HLB-resistant *C. autralasica* plants with ‘*Ca*. L. asiaticus’ [8]. To that end, a bioinformatic comparative analysis of RNASeq profiling specifically for the different GASA genes was performed from the mentioned public datasets. The results obtained from this approach are depicted in Figure 5. In the specific case of BioProject PRJNA417324 [40] (carried out using ‘*Ca*. L. asiaticus’ infected leaves from *C. sinensis)*, GASA10, 11, 12, and 13 significantly increased their expression over time until the last evaluation on day 322, showing their highest levels at that point, with a significant relative expression value for GASA10 of 4.7. Likewise, BioProject PRJNA645216 [41] denotes a significative time-dependent specific increase in GASA6 and 14 up to 5 days post-infection (dpi) in HLB-infected oranges (using *Diaphorina citri* vector) when compared to uninfected negative controls. On the other hand, it is also worth mentioning that GASA8 showed the strongest downregulation effect in uninfected plants, while this did not occur in the infected ones. For BioProject PRJNA755969 [39], a non-significant trend for higher gene expression in leaves of *C. australasica* was observed for GASA8 and 14, while GASA12 showed a statistically significant increase. This last comparison may become relevant considering that the *C. australasica* belongs to a species considered to be resistant to HLB [1,5,39,42].

In summary, GASA6 (subfamily I), 8 (subfamily II), and 10 (subfamily III) present both phylogenetic and structural distances between them, sequence similarity to well-studied orthologs in *S. tuberosum*, exhibit good basal expression levels in the juvenile leaves and/or floral tissue and demonstrate differential response in infected plants compared to their negative controls and/or increased levels of expression. As can be seen in Figure 5, in the last case, some reports demonstrated the upregulation of GASA8 after infection of *C. clementina* leaf explants with *X. citri* [24] or its downregulation with ‘*Ca*. L. asiaticus’ in *C. sinensis*, while GASA6 and 10 were upregulated with ‘*Ca*. L. asiaticus’ [40,41]. In addition, it is worth noticing that the rootstock species *P. trifoliata*, known to be tolerant to HLB [1,3,5], presented high expression of GASA6, 8, and 10 in floral tissue, while the same happened with GASA6 and 10 in young leaves. Therefore, GASA6, 8, and 10 genes from *P. trifoliata* origin were selected for further evaluation of their potential antimicrobial activity by performing *in planta* experiments.

### 2.5. Promoter Analysis of Citrus GASA6, 8, and 10 Selected Genes

SNAKIN/GASA peptides are not only known for playing an important role in response to pathogens but also display diversified biological activities in many aspects of plant growth and the development process that influence their relative expression.

The promoter regions of the selected genes from *P. trifoliata* were analyzed to identify specific cis-regulatory elements that may explain the above observed expression patterns. Thus, the putative promoter sequences from GASA6, GASA8, and GASA10 from *P. trifoliata*, *C. limon*, and *C. sinensis* were analyzed using the database of Plant Cis-Acting Regulatory DNA Elements (PlantCARE) to predict their cis-regulatory elements (Supplementary Table S2).

Several abiotic stress-related cis-regulatory elements were identified, with those putatively involved in light responsiveness (GATA-motif, GA-motif, ATCT-motif, TCT-motif, I-box, G-box, Box 4, AE-box, LAMP-element, GTGGC-motif, AF1 binding site, chs-CMA2a, GT1-motif) being clearly prevalent. This cis-regulatory element composition is expected for leaf and floral tissues. Some other motifs found are hormone-related, like the methyl jasmonate-responsiveness elements (TGACG-motif, CGTCA-motif). They were found in all analyzed genes, except for GASA6 and GASA10 in *C. limon*. An ABA-responsive element (ABRE) involved in the abscisic acid response was also present in all analyzed genes, except in GASA8 of *C. limon* and *P. trifoliata* or in GASA10 of *P. trifoliata*. Another number of cis-regulatory elements associated with gibberellin-responsive elements (TATC-box, P-box), auxin-responsive elements (AuxRR-core), were also observed. Interestingly, a salicylic acid-responsive element (TCA element) was identified exclusively in the promoter of GASA10 gene from *P. trifoliata* (among all the genes analyzed in this paper).

Since salicylic acid plays a master role in host–pathogen interactions, this distinctive presence may have an eventual connection to the upregulation of *Pt*GASA10 gene upon HLB infection described in the section before. In the same way, in some of the GASA genes analyzed, other cis-regulatory elements were found, such as environmental response factors like low temperature response (LTR) and anaerobic induction. Likewise, elements involved in expression/differentiation of some tissues (palisade mesophyll cells, meristem, endosperm cells) were also found and may contribute to the tissue-specific expression patterns observed in Figure 4. For example, GASA10 promoters of the three *Citrus* species showed a putative meristem-specific cis-regulatory element. The same occurs for GASA8, which contains a leaf mesophyll-specific element. GASA8 and GASA10 also possess MYB-binding sites putatively involved in flavonoid biosynthetic genes, drought-inducibility (MBSI and MBS), and light responsiveness (MRE).

These results suggest that GASA genes are potentially involved in development processes and multiple abiotic stress responses, consistent with observations reported in other plant species. Except for the mentioned tissue-specific regulatory elements, which may explain the differences in basal gene expression levels between young and mature leaves or between flower tissues and leaves shown in Figure 4, and for the salicylic acid-responsive motif present in *P. trifoliata* GASA10 promoter, no highlighting differences were detected in the promoter sequences when homologous GASA genes from the three species were compared.

### 2.6. Overexpression of PtGASA6 and 10 Decreased Disease Symptoms Caused by Pseudomonas syringae pv. tabaci

**Figure 6:**
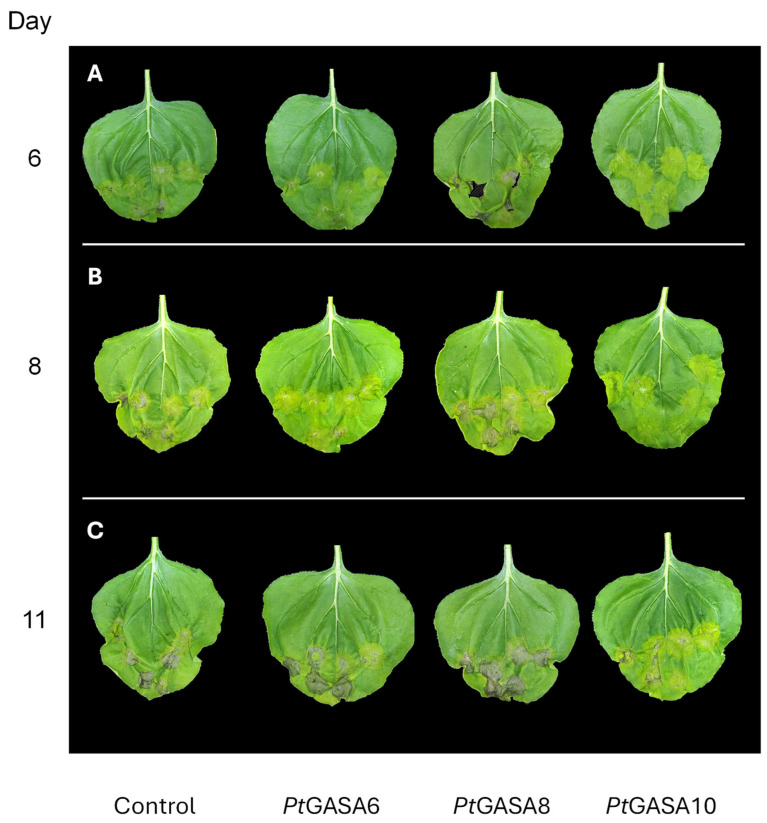
Illustrative photo ensembles of individual representative leaves of *N. benthamiana.* Leaves were infected with *P. syringae* pv. *tabaci* transiently overexpressing *Pt*GASA6, *Pt*GASA8, and *Pt*GASA10 or the control at 6 (**A**), 8 (**B**), and 11 (**C**) days post-infection. Each leaf was co-infiltrated in the same six positions, with *A. tumefaciens* GV3101 expressing the indicated genes and the pathogen *P. syringae* pv. *tabaci*.

To assess the antimicrobial effect of selected GASA genes, an *in planta* assay based on *Nicotiana benthamiana* agroinfiltration was employed. This species was chosen because it is a well-established model for transient gene expression, is susceptible to several pathogens and has a fully characterized genome. Overexpressions of *Pt*GASA6, 8, and 10 genes were significant (*p*-value < 0.001) in agroinfiltrated leaves containing the corresponding gene constructs. *Pt*GASA6, 8, and 10 showed 9.28-, 6.72-, and 5.78-fold increases in expression relative to the control, respectively (Supplementary Figure S3). A disease assay was then performed by inoculating *N. benthamiana* with *P. syringae* pv. *tabaci*, a pathogen of this plant species. To accomplish this, three independent co-infiltrations with *Agrobacterium tumefaciens* expressing *Pt*GASA6, *Pt*GASA8, or *Pt*GASA10 were performed or with *A. tumefaciens* harboring an empty Ti plasmid as the negative control. Each leaf was infiltrated at six identical positions across 15 biological replicates (see Figure 6).

**Figure 7:**
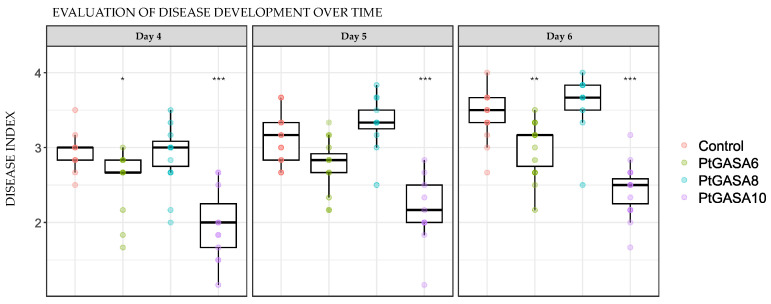
Time course of disease index progression. Evaluation of disease development on days 4, 5, and 6 post-challenge with *P. syringae* pv. *tabaci* in leaves overexpressing the genes *Pt*GASA6, *Pt*GASA8, and *Pt*GASA10, or control. The non-parametric statistical analysis was performed using the Wilcoxon rank-sum test with continuity correction, adjusting *p*-values by the Bonferroni method relative to the control. Statistical significance is indicated by asterisks as follows: * *p* < 0.05, ** *p* < 0.01, *** *p* < 0.001.

The development and progression of necrotic disease symptoms in individually infiltrated leaf circles were monitored for 7 dpi. Disease symptoms were measured using a Disease Index (DI) adapted from Schandry et. al. [43]. As a result, the overexpression of *Pt*GASA6 and 10 significantly reduced symptom development compared to *Pt*GASA8 or to the negative control. This reduction was especially fast and conspicuous for *Pt*GASA10 (Figure 7).

**Figure 8:**
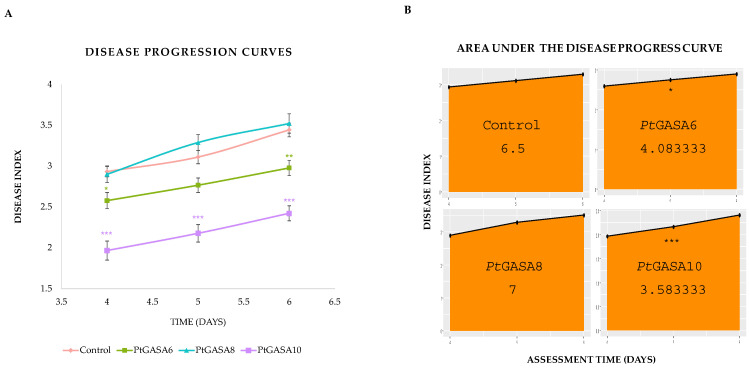
Time course of disease AUDPC progression. Evaluation of disease development caused by *P. syringae* pv. *tabaci* in leaves overexpressing *Pt*GASA6, *Pt*GASA8, and *Pt*GASA10 or control. (**A**) Disease progression curves showing the disease index over time for *Pt*GASA6, *Pt*GASA8, *Pt*GASA10, and control. It is represented in green, light blue, purple, and magenta, respectively. (**B**) Area under the disease progress curve for the control and *Pt*GASA6 (**upper** panel), *Pt*GASA8, and *Pt*GASA10 (**lower** panel), respectively. Statistical analysis was performed using the non-parametric Wilcoxon rank-sum test with continuity correction and Bonferroni adjustment relative to the control. Significance levels are indicated by asterisks: * *p* < 0.05, ** *p* < 0.01, *** *p* < 0.001.

As the disease progressed, symptoms severity was monitored over time, and leaves expressing *Pt*GASA6 or *Pt*GASA10 presented much milder symptoms than those expressing *Pt*GASA8 or the negative control, with *Pt*GASA10 showing the greatest delay in disease progression (Figure 7). These data were subsequently used to calculate the area under the disease progress curve (AUDPC), revealing that *Pt*GASA6 and especially *Pt*GASA10 showed a significant reduction in AUDPC, indicating that the decrease in symptoms relative to the control persisted throughout the evaluation period (Figure 8).

### 2.7. Overexpression of PtGASA6 and 10 Increased Hypersensitive Response (HR) After Leaf Infiltration with Xanthomonas citri

Since disease symptom progression caused by *P. syringae* infecting *N. benthamiana* was retarded when *Pt*GASA6 or 10 were overexpressed, a possible role in defense could be attributed to these genes. To confirm this hypothesis, an *in planta* HR assay was conducted by inducing this response through syringe infiltration of 1 × 10^7^ CFU/mL of *X. citri* in *N. benthamiana*. This experiment provides a contrasting scenario between a typical incompatible host–pathogen interaction and the compatible interaction analyzed in the previous section, in the well-known *N. benthamiana* model. Although *X. citri* induces an HR response in *N. benthamiana*, it is a highly pathogenic bacterium in *Citrus* varieties [44,45], making it interesting to analyze these results in connection with potential *Citrus* defense mechanisms. Since disease symptom progression caused by *P. syringae* infecting *N. benthamiana* was retarded when *Pt*GASA6 or 10 were overexpressed, a possible role in defense could be attributed to these genes.

**Figure 9:**
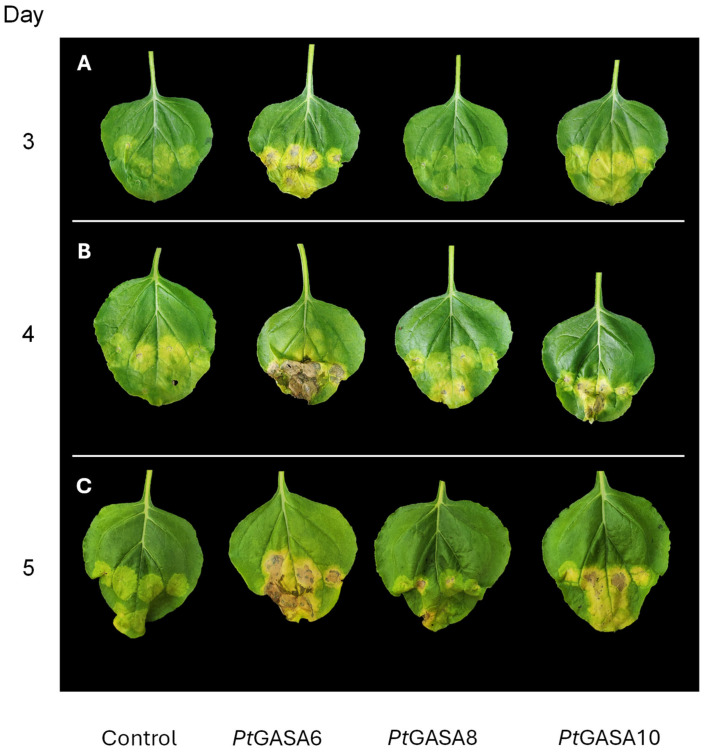
Demonstrative photograph ensemble of representative individual leaves of *N. benthamiana* after transient overexpression of *Pt*GASA6, *Pt*GASA8, and *Pt*GASA10 (or the control) showing the hypersensitive response generated by challenging with *X. citri* subsp. *citri* at 3 (**A**), 4 (**B**), and 5 (**C**) days post-infection.

**Figure 10:**
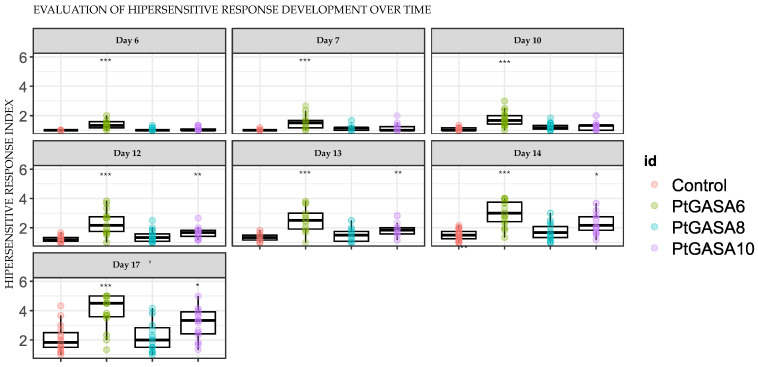
Time course development of hypersensitive response, from days 6 to 17, post-challenge. The leaves overexpressing the genes *Pt*GASA6, *Pt*GASA8, and *Pt*GASA10 or the control were inoculated with *X. citri* subsp. *citri*. The overexpressing leaves and control are represented in green, light blue, purple, and magenta, respectively. The non-parametric statistical analysis was performed using the Wilcoxon rank-sum test with continuity correction, adjusting *p*-values by the Bonferroni method relative to the control. Statistical significance is indicated by asterisks as follows: * *p* < 0.05, ** *p* < 0.01, *** *p* < 0.001.

**Figure 11:**
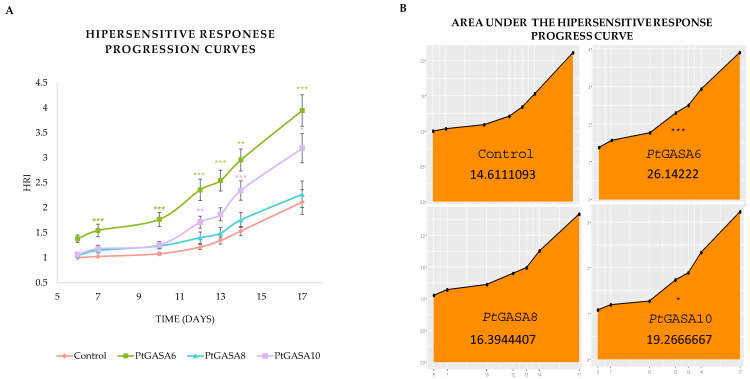
Evaluation of hypersensitive response (HR) development caused by *X. citri* subsp. *citri. X. citri* infected leaves overexpressing *Pt*GASA6, *Pt*GASA8, and *Pt*GASA10 or control. (**A**) Hypersensitive response progression curve using the HR index (HRI) as a function of time. The overexpressing leaves and control are represented in green, light blue, purple, and pink, respectively. (**B**) Area Under the hypersensitive response progress curve (AUHRPC). Non-parametric statistical analysis was performed using the Wilcoxon rank-sum test with continuity correction, and *p*-values were adjusted by the Bonferroni method relative to the control. Statistical significance is indicated by asterisks as follows: * *p* < 0.05, ** *p* < 0.01, *** *p* < 0.001.

A significantly higher induction of HR symptoms was observed upon overexpression of *Pt*GASA10 and, especially, *Pt*GASA6 (Figure 9 and Figure 10). In contrast, overexpression of *Pt*GASA8 did not result in consistent significant differences compared with the negative control among both repetitions. The disease symptoms were quantified using an HR index (Figure 11). Each of the six infiltrated leaf circle positions was individually monitored across all leaf replicas at 14 dpi for visible HR symptoms (Figure 8B). *Pt*GASA10 and particularly *Pt*GASA6 presented a meaningfully increased HR response trend (Figure 11).

**Figure 12:**
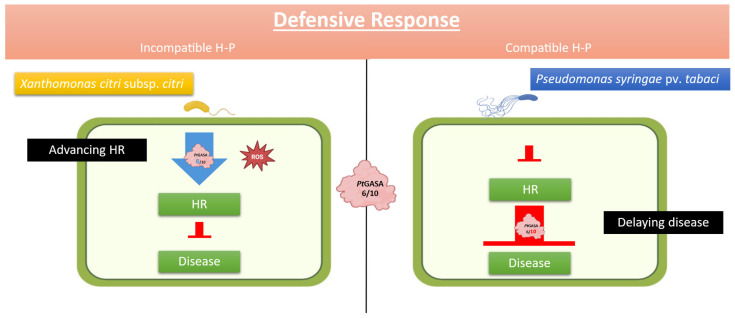
An illustrative model representing the events observed during infections. *Pt*GASA6 and *Pt*GASA10 were overexpressed under incompatible pathosystem with *X. citri* subsp. *citri* (**left** panel) and compatible interaction with *P. syringae* pv. *tabaci* (**right** panel). For both genes, an earlier hypersensitive response was observed, as well as a delayed onset of necrosis development because of the disease. A stronger enhancement was observed for *Pt*GASA6 in advancing hypersensitive response and *Pt*GASA10 in delaying disease.

Interestingly, *Pt*GASA6 enhanced the hypersensitive response by 30% more than *Pt*GASA10, whereas *Pt*GASA10 delayed necrosis by 40% more than *Pt*GASA6. These contrasting results suggest that the mode of action of these proteins is different (Supplementary Table S3 and Figure S4). Collectively, the HR and disease assay results suggest that immunity may be heightened through rapid priming of an HR, potentially sustained over time, as indicated by the consistent trend line observed during GASA overexpression, which remains relatively stable. Therefore, a reduction in disease symptoms is observed. However, an additional or parallel mechanism may also be involved. This is supported by the observation that *Pt*GASA6 exhibited statistically significant higher DIs and AUDPCs in comparison to *Pt*GASA10, but also a more pronounced HR index and area under the hypersensitive response progress curve. As detailed in the Section 3, we propose a model to illustrate these differences (Figure 12).

### 2.8. Structural and Funtional Comparison Between Predicted Tridimensional Structures of Citrus GASA Proteins Versus Reference SNAKIN Proteins from Solanum tuberosum

**Figure 13:**
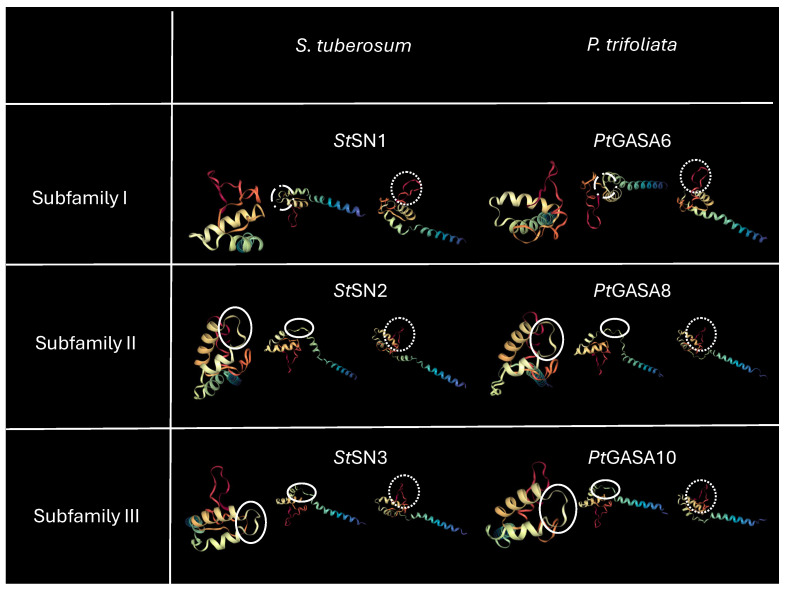
Schematic representation of conserved motifs in subfamilies I, II, and III of the SNAKIN/GASA family from *S. tuberosum* (**left**) and *P. trifoliata* (**right**). The first row corresponds to subfamily I, comprising *St*SN1 and *Pt*GASA6; the second to subfamily II, including *St*SN2 and *Pt*GASA8; and the third to subfamily III, with *St*SN3 and *Pt*GASA10. The motif common to all subfamilies and species (*P. trifoliata* and *S. tuberosum*) is indicated by a dotted line (·······). The motif characteristic of subfamily I, shown with a dash-dot line (--·--), confers a more compact arrangement with condensed α-helices. In contrast, the motifs present in subfamilies II and III, represented by a solid line (──), promote a looser structure with more separated α-helices.

It was reported that the ability of plant AMPs to act against a large spectrum of pathogens depends on their structural properties [11,23]. For this reason, a comparative evaluation of the tertiary structure of the selected peptides was performed using the Alphafold2 tool [46] and MMseqs2 [47] in the Colab v1.5.5 environment [48,49] to generate their structural visualization. The structures were then visualized using the NGL Viewer tool version 2.0 [50]. As can be observed in Figure 13, all proteins analyzed are predicted to adopt a helix-turn-helix (HTH) motif as well as random coil folding motifs. A common motif (denoted with a dotted line ·······) presented an identical conformation across all members of the three subfamilies and is identical in both species being compared, *P. trifoliata* and *S. tuberosum*. Crystallography studies of *St*SN1 demonstrated that this specific motif corresponds to the turn between the two helices of the HTH [51]. Although the three subfamilies seem to share other common motifs, it was noticed that those indicated with a solid line show greater similarity between subfamilies II and III, since they generate a looser arrangement of the α-helixes, which appear more separated. On the other hand, the characteristic motif of subfamily I, marked with lines and dots (-- · --), results in a more compact structure, with more condensed α-helixes.

**Figure 14:**
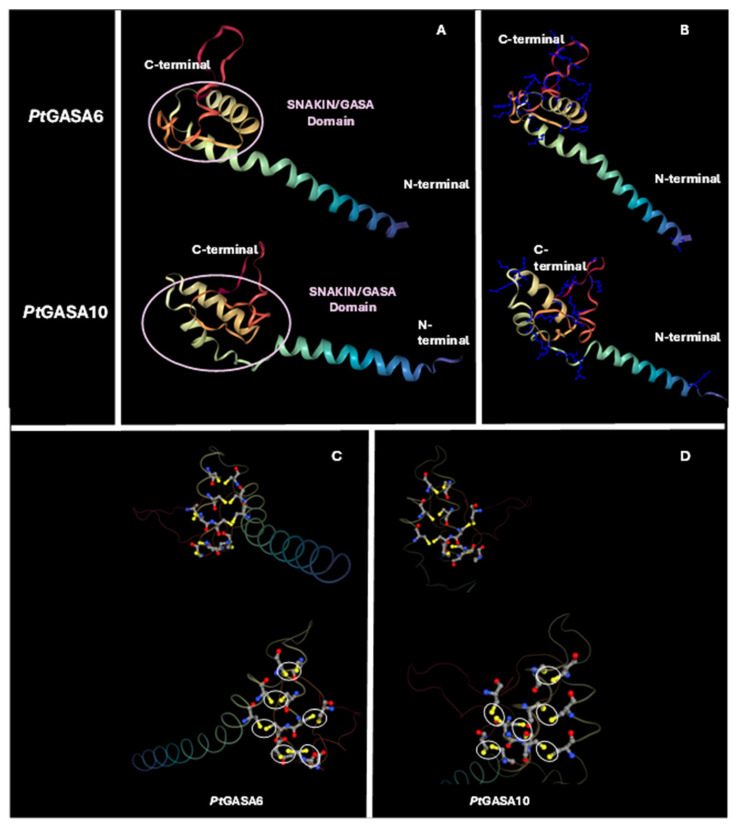
Three-dimensional structure of *Pt*GASA6 and *Pt*GASA10. (**A**) The C-terminus and N-terminus and the location of the SNAKIN/GASA domain are indicated. (**B**) Positively charged amino acids (arginine, lysine, or histidine) are shown in blue, mainly located within the HTH region. (**C**) Three-dimensional structure of *Pt*GASA6 and (**D**) *Pt*GASA10 showing the 6 in silico–disulfide bonds predicted. Cysteine residues are highlighted in yellow. The predicted bonds are indicated by white circles.

**Figure 15:**
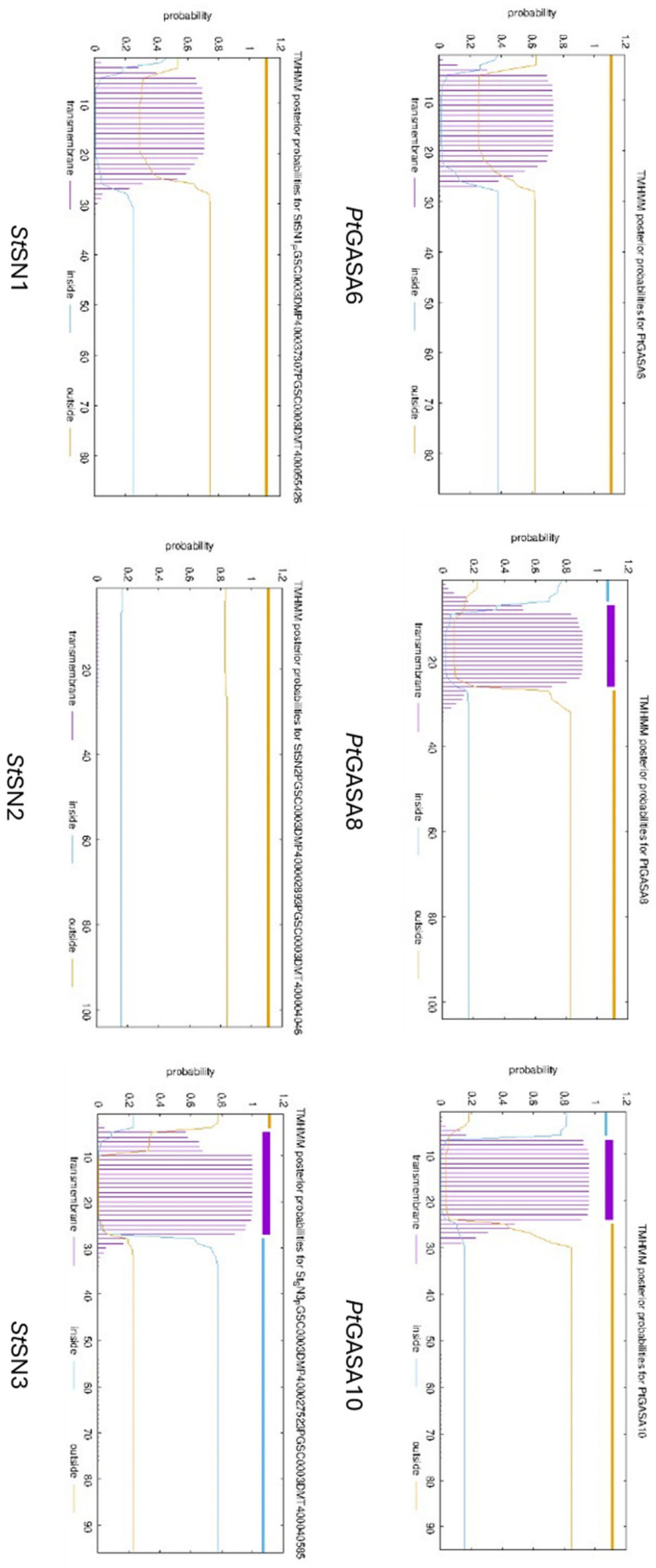
Topology of proteins *Pt*GASA6, *Pt*GASA8, *Pt*GASA10, *St*SN1, *St*SN2, and *St*SN3. The purple line indicates the probability that a segment corresponds to a transmembrane affinity region. The light blue line at the top of the plot represents the probability regions are oriented toward the cell interior (cytoplasmic). The yellow line marks the regions probably located on the outer side of the cell (extracellular). The predicted topology suggests that *Pt*GASA8 and *Pt*GASA10 may contain a membrane-anchoring site (transmembrane region).

As described by Nahirñak et al. [30], the SNAKIN/GASA proteins have three characteristic domains: (1) at the N-terminal end is the peptide signal; (2) an intermediate region; (3) at the C-terminal end is the SNAKIN/GASA motif, which includes the conserved aminoacidic positions that give the name to this family of proteins, such as the array of 12 cysteines responsible for the structural stability of the peptide, as well as variable regions that allow them to be classified into the three previously mentioned subfamilies. In full agreement with these conserved domains, Figure 14 shows that tertiary structures of the *Pt*GASA6 and *Pt*GASA10 proteins follow this architectural scheme. The HTH region has a large proportion of positively charged amino acids, such as arginine, histidine, and lysine, in accordance with what has been described for *St*SN1, which could be indicative of their possible mechanism of action. Some authors point out that, in addition, structurally conserved α-helixes must also be analyzed as an essential part of understanding the mechanism of action of proteins with AMP activity. In this context, a distinctive feature investigated is the presence of transmembrane domains that may help to predict differential subcellular targets like the apoplast, cellular membrane, or cell wall. To better understand this potential relationship between GASA protein structures and their putative antimicrobial mechanism, an in silico search of *Pt*GASA6, 8, and 10 proteins was carried out using the Deep-TMHMM algorithm (Prediction of Transmembrane Helices in Proteins) [52] and compared to their closest potato orthologs (for some of which subcellular localization is available). *Pt*GASA6, like *St*SN1, both belonging to subfamily I [23,24,30] do not appear to contain an anchoring site in the plasma membrane (Figure 15). It was reported that *St*SN1 interacts with *St*SUT1, a protein that targets phloem tissue [23] and the same could be true for *PtGASA6* but must be investigated. On the contrary, *Pt*GASA8 and *Pt*GASA10 present at least one α-helix motif in their signal peptides with putative binding properties to membrane structures, suggesting they may either remain anchored or be secreted. These results are also consistent with those observed in *C. clementina* [24]. Although these predicted structural differences that suggest different subcellular targets between *Pt*GASA6 and 10 certainly require subcellular localization experiments for validation, these predictive data are compatible with our belief that their defensive mechanisms are somewhat different too.

## 3. Discussion

By leveraging a pan-genomic framework, this study expands the catalog of citrus GASA genes with 67 new curated variants across rootstock-enriched germplasm. The phylogenetic relationship among all GASA proteins showed that members of each subfamily grouped together, even when they belong to evolutionary distant species. These results indicate that, despite the important nucleotide sequence diversity evidenced, the three core subfamilies already found in other plant species, are conserved in *Citrus* germplasm as well [18,24,30,53,54]. This phylogenetic clustering leads to the hypothesis that defense mechanisms conferred by *Citrus* GASA genes may also follow different possible modes of action, according to the subfamily they belong to, as assumed in the studies described in Section 2.5 and Section 2.6.

Previous studies by Wu et al. [24,31] demonstrated that several GASA genes exhibit basal expression in different tissues of *C. clementina*. In agreement with these findings, these results across rootstock accessions show that juvenile leaves and floral tissues display higher GASA expression than mature leaves, a pattern coherent with developmental roles and defense priming in actively growing tissues [11,23,24,30,55]. Wu et al. [24] also investigated the comparative expression of GASA genes after infections with *X. citri* in detached leaf explants of *C. clementina*. From these experiments, the authors conclude that six genes, including GASA8, presented a significant increase in expression. The induction of these genes could indicate that, although they have a good basal expression in the tissues where the *X. citri* infection is produced, their expression increases after infection; so, these genes could be playing some role against it [24] or be a collateral effect of the infection process. Moreover, meta-analysis of HLB-related RNA-seq datasets revealed upregulation of GASA10–13 in infected *C. sinensis* over extended time courses, as well as early induction of GASA6 and GASA14 upon psyllid feeding that transmits ‘*Ca*. L. asiaticus’ [40,41]. Conversely, GASA8 exhibited contrasting behavior—repression in some uninfected conditions, but induction in *C. clementina* explants challenged with *X. citri*—highlighting context dependence across host–pathogen settings [24,39,40]. A final indication of specific gene induction by pathogens comes from *S. tuberosum*, where *St*SN3 (a potato ortholog of GASA10) is upregulated by *P. syringae* pv. *tabaci* [18,30]. *P. syringae* is the very same pathogen that was used in this paper to challenge GASA10 overexpression in *N. benthamiana*.

In addition, promoter mining provided a mechanistic bridge hypothesis to these patterns. Specifically, all three selected genes contain light-responsive elements (consistent with leaf/flower expression) and ABA/MeJA motifs (abiotic stress and defense). Most importantly, *Pt*GASA10 exhibited a unique salicylic acid (SA)-responsive TCA element among the analyzed promoters reported in the literature [19,23,24,30]. Given SA’s central role in biotrophic pathogen defense and HR signaling, this cis-signature plausibly could underpin *Pt*GASA10’s potential behavior under HLB-related contexts and its effect on delaying symptom development [39,40]. In contrast, *Pt*GASA6 lacked a predicted membrane anchor and SA motif, pointing toward a different contribution mechanism to HR priming and signaling [18,23,24,30,55]. The fact that some of the members are induced by infection while others are not show that some members adjust to the standard basic definition of acquired passive resistance (they are ‘just there’, passively waiting to prevent infection without any changes), while others are actively induced by the infection process. All together, these results suggest that, among the wide pan-genomic diversity of GASA genes, more than one single mechanism of resistance may coexist within this large gene family.

To prove that any of the new genes/alleles really confer resistance to bacterial diseases, it was designed for functional validation by using a tractable pathosystem, i.e., by performing transient expression assays in *N. benthamiana.* As a result, *Pt*GASA6 and *Pt*GASA10 enhanced defense responses through distinct mechanisms. *Pt*GASA6 accelerated hypersensitive response (HR), while *Pt*GASA10 delayed necrosis caused by *P. syringae* pv. *tabaci*. *Pt*GASA6 and 10 complementary effects suggest subfamily-specific roles in pathogen resistance [11,23,24,55] (see Figure 7, Figure 8, Figure 9, Figure 10 and Figure 11). The underlying mechanisms still need a thorough molecular approach to better understand how GASA proteins fit in the complex induction of the HR system and how they specifically interact with its molecular components but clearly open a door. The elucidation of the detailed molecular mechanism exceeds the scope of this report.

By contrast, *Pt*GASA8—despite expression evidence in tolerant rootstocks—did not show any significant differences from the negative control in triggering HR in *N. benthamiana* leaves when challenged with *P. syringae* or *X. citri* [24,39]. In other words, *Pt*GASA8 did not yield consistent phenotypic benefits under our specific conditions, underscoring the gene and context specificity of AMP-mediated defense. As explained, *Pt*GASA8 belongs to a different subfamily and has structural differences from the other two and to its homolog protein in potato Snakin2, as can be observed in Figure 13. Not all GASA proteins may have antibacterial activity or include participation in HR as part of their resistance mechanism; its specific induction in other systems could be a collateral effect of the infection process or display other defense mechanisms alternative to HR enhancement.

In conclusion, these findings confirm that at least two GASA genes confer measurable resistance traits in vivo. Together, these results echo and extend past findings in potato: some members of the snakin family are inducible by pathogens and can curtail disease spread, but their net outcome depends on subfamily identity, expression level, and subcellular localization [18,23,24,55]. SNAKIN/GASA genes enhance defense against pathogens by a variety of mechanisms. Enhancement of hypersensitive response and necrosis reduction could be just two of them, and maybe, GASA8 has others. Importantly, in this experiment, *Pt*GASA10 delayed necrosis more robustly than *Pt*GASA6 despite lower expression, suggesting higher functional efficiency, distinct kinetics, or greater protein stability in the infection milieu.

Furthermore, these agroinfiltration assays demonstrate that overexpression of *Pt*GASA6 or *Pt*GASA10 effectively triggered HR after *X. citri* infection and significantly reduced symptoms caused by *P. syringae* pv. *tabaci*. In contrast, these effects were absent at basal expression levels in the negative control. Consequently, the mechanism appears to be expression-dependent for these two genes.

Regarding whether the observed pan-genomic diversity is shaped, at least partially, by coevolutionary dynamics with specific pathogen species (analogous to classical gene-for-gene interactions) or it largely reflects neutral evolutionary processes and redundancy within the pangenome, most of the evidence collected to date in the literature and in this paper suggests that both could be true. Comparison between potato and citrus predicted protein structure of the mentioned protein members representing subfamilies I, II, and III in potato and citrus evidenced a strong conservation of the tertiary structure. Despite the large phylogenetic distance between potatoes and citrus, they consistently predict the helix-turn-helix (HTH) fold with cysteine-rich motifs fundamental for antimicrobial peptide (AMP) stability and activity [18,23,30]. However, while for *St*SN1/GASA6 and *St*SN3/GASA10 comparison, only minor differences were noticed, for *St*GASA8, a novel signal peptide with predicted transmembrane properties is present when compared to *St*SN2, suggesting a major change in subcellular destination and that significative evolutionary changes occurred even within the same gene orthologs of subfamily II. In other words, despite cross-species conservation, subfamily-specific structural signatures were detected (i.e., compact α-helices in subfamily I vs. looser arrangements in II/III) [10,18,24,30,53], consistent with potential differences in target interaction and mode of action and suggests functional partitioning along evolutionary lines [18,23,24,26,51,56,57,58,59]. Taken together, structural and phylogenetic evidence suggests that both processes may be involved. On the one hand, orthologous pairing to reference potato snakin proteins revealed strong conservation of tertiary structure despite large phylogenetic distance, indicating selection for antimicrobial peptide stability. On the other hand, high allelic and paralog diversity—exemplified by GASA9/GASA9-like—points to neutral drift and sub-functionalization.

These differences could indicate that the mechanisms of action are either different or that they are involved in alternative steps of metabolic pathways. Although specific dose–response studies are needed to draw conclusions, it is noteworthy that *Pt*GASA10 had a stronger effect in delaying disease necrosis despite its lower expression level (twice as low as that of *Pt*GASA6). This suggests that *Pt*GASA10 possesses the remarkable ability to slow down the progression of the disease, even with reduced expression, which may indicate a high functional efficiency, a particularly effective mechanism of action or that it has a longer protein lifetime. In this context, it is noteworthy to mention that *P. syringae* pv. *tabaci* induces the expression of *St*SN3 [18], which is an orthologue of *Pt*GASA10. This could be an indication that, during infection caused by bacteria, the plant may use this protein to prevent the systemic spread of bacteria in plant tissues.

As mentioned before, *Pt*GASA6 lacks the membrane anchoring site observed for *Pt*GASA10 structure, suggesting that it could play its defensive role in a different cellular compartment, where it leads to the induction of a stronger HR compared to *Pt*GASA10. The evolutionary dichotomy is consistent with the subfamily-dependent structural constraints we observed (HTH packing differences) and with literature reports of pleiotropy, where SNAKIN/GASA proteins balance developmental roles with immune activation [10,19,22,23,26]. Regarding the mechanism involved, we propose a working model in which *Pt*GASA6 acts as an early amplifier of HR, likely through cellular pathways that escalate ROS bursts, ion fluxes, and localized cell death at infection foci—key components of incompatible responses [23,26,30]. Conversely, *Pt*GASA10—harboring a predicted membrane targeting signature and a SA-responsive promoter element—appears to retard lesion expansion and limit tissue damage over time. It might operate at the cell wall–apoplast interface, attenuating bacterial colonization, neutralizing extracellular factors, or modulating cell wall integrity pathways; such localization would also enhance direct contact with pathogen cells during early invasion [23,24,55]. However, these speculations based on predictive models (i.e., structural and consensus cis-elements discovery) deserve a note of caution before making conclusive arguments because they must be corroborated experimentally, since discrepancies could occur [30].

The results reported may have translational implications for citrus improvement. From a breeding and biotech perspective, *Pt*GASA6 and *Pt*GASA10 emerge as complementary levers: one to reinforce HR priming (*Pt*GASA6) and another to suppress disease progression (*Pt*GASA10). Practical routes include rootstock engineering (overexpression or promoter editing) to confer broad-spectrum protection versus scion modification [2,3,14], CRISPR-based promoter modulation (CRISPR motif editing) to recreate SA/MeJA responsiveness and mobilize defense only under challenge, mitigating pleiotropy [60,61,62,63], allele mining to discover naturally optimized variants with favorable efficacy–growth trade-offs [64,65]. Given the demonstrated sexual compatibility within *Citrus* and the recognized HLB tolerance in *P. trifoliata*, marker-assisted introgression or cisgenic approaches using *Pt*GASA alleles provide realistic avenues toward field-relevant resilience [1,3,4,5].

## 4. Materials and Methods

### 4.1. Plant Material and Bacterial Strains

Citrus plant material was collected from the experimental germplasm bank orchard at EEA INTA Bella Vista (Corrientes Province, Argentina), including *Citrus sinensis* (sweet orange), *C. limon* (lemon), Citrumelo (*Citrus* × *paradisi* × *Poncirus trifoliata*) and EEA INTA Concordia (Entre Ríos Province, Argentina) including *C. jambhiri* (rough lemon), *C. limonia* (Rangpur), *C. warburgiana* (New Guinea wild lime), *C. aurantium* (bitter orange), and *P. trifoliata* (trifoliate orange). Fully developed trees were used as the tissue source for gene cloning, gene expression analysis, and template origin for transient expression assays.

*Nicotiana benthamiana* model system was used for agroinfiltration and infection assays. Plants were grown in controlled growth chambers under 22–25 °C, with a 16 h light and 8 h dark photoperiod.

*P. syringae* pv. *tabaci* and *X. citri* subsp. *citri* isolates were used for infection assays. Bacterial suspensions were prepared from 24–48 h cultures in King’s B medium (*P. syringae*) or nutrient agar (*X. citri*) and adjusted to OD_600_ as expressed in Section 4.6.

### 4.2. Identification of GASA Genes/Allelic Variants, PCR Cloning, and DNA Sequencing

Genomic and coding sequences (CDSs) of *Citrus* GASA genes were retrieved from the Citrus Genome Database [66] and NCBI [67]. DNA was extracted from citrus leaf tissue using a standard CTAB method [68]. DNA quality and concentration were measured using a NanoDrop 2000C spectrophotometer (Thermo Fisher Scientific, Waltham, MA, USA).

PCR amplifications were performed using oligonucleotides designed to amplify conserved GASA family sequences (Supplementary Table S4). PCR products were cloned into pGEM-T Easy vector (Promega, Madison, WI, USA), transformed into *Escherichia coli*, and sequenced with Applied Biosystems^TM^ universal phage M13 forward and M13 reverse primers (Waltham, MA, USA). Three independent clones were sequenced per product. In total, 67 curated genomic variants were obtained from 16 amplified *loci* and deposited in GenBank (accessions: OP728335 to OQ053292; Table 1).

All gene variants presented a signal peptide sequence and the SNAKIN/GASA domain, which includes the diagnostic signature of 12 cysteines in highly conserved positions. By using this information, a bioinformatic analysis was carried out using the NCBI [67,69] and *Citrus* Genome databases [66], in which the amino acid consensus sequence was used to identify the corresponding gene and obtain the probable identifiers of this family of proteins (see Table 1 and Figure 1).

### 4.3. Structural and Phylogenetic Analyses of GASA Sequences

Protein sequences were aligned using Clustal Omega version 1.2 [70]. The presence of conserved GASA domains was confirmed using JPred version 4 [71], and signal peptides were predicted with SignalP 5.0 [72].

A phylogenetic dendrogram was constructed using the Neighbor-Joining method in MEGA11 [73], with bootstrap support from 1000 replicates. Evolutionary distances were calculated using the Poisson correction model [74], based on 83 amino acid sequences (194 aligned positions after pairwise deletion).

GASA gene chromosomal positions were mapped using genome data from *C. sinensis*, *P. trifoliata* and *C. limon* of Citrus Genome Database [66], and graphic represented using MapChartversion 2.2 [75]. For promoter region analysis, 1500 bp DNA sequences upstream of the ATG site were extracted from Citrus Genome Database [66] and the putative cis-regulatory elements were determined with plantCARE database [76] and classified with TBTools [77].

### 4.4. Expression Analysis by RT-qPCR

Total RNA was extracted from citrus and *N. benthamiana* tissues using TransZol reagent (TransGen Biotech, Beijing, China), following the manufacturer’s instructions. RNA integrity and purity were verified by agarose gel electrophoresis and spectrophotometry (NanoDrop 2000C, Thermo Fisher Scientific, Waltham, MA, USA). Genomic DNA was removed by DNase I treatment (Thermo Scientific, Waltham, MA, USA), and cDNA synthesis was carried out using M-MLV Reverse Transcriptase (Thermo Scientific, Waltham, MA, USA) with random hexamer oligonucleotides and 1 µg of total RNA.

Quantitative PCR reactions were performed using Platinum™ Taq DNA Polymerase (Invitrogen, Waltham, MA, USA), dNTPs (200 µM each), 3 mM MgCl_2_, ROX passive reference dye (Thermo Fisher Scientific, Waltham, MA, USA), and SYBR Green I (Thermo Fisher Scientific, Waltham, MA, USA) in a final volume of 10 µL. Reactions were run on a StepOnePlus PCR System (Applied Biosystems, Waltham, MA, USA).

For the analysis of GASA gene expression in citrus tissues, an absolute quantification approach was employed. Gene-specific oligonucleotides were used for each GASA and CsGAPC2 (glyceraldehyde-3-phosphatedehydrogenase 2) [78] as reference for normalization. Standard curves were generated using serial dilutions of purified PCR amplicons cloned for each of the selected GASA genes. These curves were used to determine the absolute copy number of transcripts per ng of cDNA for each gene in floral, young leaf, and mature leaf tissues from five different citrus accessions.

To evaluate gene expression following transient agroinfiltration in *N. benthamiana*, a relative quantification method was used. Gene expression levels of *Pt*GASA6, *Pt*GASA8, and *Pt*GASA10 were calculated using the comparative Ct method (ΔCt) [79], normalized to an internal reference (ubiquitin 3), and expressed as log-2-fold change relative to leaves infiltrated with an empty vector control. Three biological replicates were analyzed per gene construct, and results are represented as mean ± standard errors. Statistical analysis of overexpression was conducted using the FgStatistics package, version 2012 [80] with paired technical replicates and 5000 bootstrap resampling cycles. Heatmap was created with the ChiPlot online tool, version 2.6.1 [81].

### 4.5. Transcriptomic Meta-Analysis of Citrus GASA Gene Expression in Public RNA-Seq Datasets

Ten publicly available BioProjects were retrieved from the NCBI database that investigate gene expressions in citrus under huanglongbing (HLB) infection conditions as follows: PRJNA348468, PRJNA417324, PRJNA574168, PRJNA629966, PRJNA640485, PRJNA645216, PRJNA739184, PRJNA739186, PRJNA755969, and PRJNA780217 (Supplementary Table S1). These datasets include multiple tissues, genotypes, and time-points post-infection and were selected following the meta-analytic framework described by Machado et al. [7] for large-scale citrus HLB transcriptome integration.

FASTQ files were processed for quality control with FastQC and trimmed for adapters and low-quality sequences using Trimmomatic, version 2025 [82]. Filtered reads were aligned to the *C. clementina* genome v1.0 using STAR v2.7.8a [83] and gene counts obtained with feature Counts v2.0.1 [84]. Read counts per sample were normalized using DESeq2 v1.34.0 [85] to correct for library size differences. Differential gene expression analyses (DESeq2) were run for each experimental comparison within each BioProject, and genes with adjusted value < 0.05 were considered differentially expressed.

To focus on the SNAKIN/GASA family, we filtered count data by the following *C. clementina* loci identifiers as follows: Ciclev10017244m.v1.0 (GASA 1), Ciclev10022925m.v1.0 (GASA 2), Ciclev10023012m.v1.0′ (GASA 3), Ciclev10033135m.v1.0 (GASA 4), Ciclev10033115m.v1.0 (GASA 5), Ciclev10002979m.v1.0 (GASA 6), Ciclev10002796m.v1.0 (GASA 7), Ciclev10002927m.v1.0 (GASA 8), Ciclev10002984m.v1.0 (GASA 9), Ciclev10013200m.v1.0 (GASA 10), Ciclev10012786m.v1.0 (GASA 11), Ciclev10013454m.v1.0 (GASA 12), Ciclev10029695m.v1.0 (GASA 13), Ciclev10006931m.v1.0 (GASA 14), Ciclev10006668m.v1.0 (GASA 15), Ciclev10006310m.v1.0 (GASA 16), Ciclev10006347m.v1.0 (GASA 17), and Ciclev10006243m.v1.0 (GASA 18). Normalized counts of these virtual genes were extracted and visualized across genotypes, tissues, and time-points via heatmaps, enabling assessment of induction, repression, or constitutive expression trends under HLB challenge.

### 4.6. Transient Overexpression and Infection Assays in N. benthamiana

For *Agrobacterium*-mediated transient expression, the genes *Pt*GASA6, *Pt*GASA8, and *Pt*GASA10 were cloned into the Gateway^®^ binary vector pK2GW7, under the control of the CaMV 35S constitutive promoter. Constructs were verified by Sanger sequencing (UGB-INTA Castelar, Hurlingham, Argentina) and transformed into the *A. tumefaciens* strain GV3101. Cultures were grown overnight at 28 °C with shaking (200 rpm) in liquid LB medium supplemented with the appropriate antibiotics (rifampicin 100 µg/mL, gentamicin 40 µg/mL, spectinomycin 100 µg/mL) and 100 µM acetosyringone. After centrifugation (4000× *g*, 10 min), bacterial pellets were resuspended in MES buffer and adjusted to an optical density (OD_600_) of 0.6. Fully expanded leaves of 2-month-old *N. benthamiana* plants were infiltrated using a needleless syringe. Infiltrations were performed in six marked positions per leaf to allow positional normalization of symptom development. For disease progression assay, the pathogenic strain *P. syringae* pv. *tabaci* was used to challenge *N. benthamiana* plants. Bacterial cultures were grown overnight at 28 °C on King’s B solid medium (KBM), washed twice with 10 mM MgCl_2_ and adjusted to a final concentration of 2 × 10^6^ CFU/mL. The final inoculum mix was prepared by combining *A. tumefaciens* and *P. syringae* pv. *tabaci* cultures at final concentrations of OD_600_ = 0.6 and 1 × 10^6^ CFU/mL, respectively. This mixture was used to co-infiltrate single leaves (one per plant), always targeting the same six leaf positions. Disease symptoms (necrosis) were recorded from 4 days post-inoculation (dpi) up to 6 dpi, blind to treatment. Fifteen plants per *Agrobacterium*-mediated transient expression gene were infiltrated. A Disease Index (DI) was assigned per leaf by averaging scores from the six infiltrated spots. Disease progression was quantified using the area under the disease progress curve (AUDPC).

For hypersensitive response (HR) assays, transiently infiltrated *N. benthamiana* leaves were challenged with *X. citri* subsp. *citri*, a non-host pathogen. *X. citri* cultures were grown overnight at 28 °C on Nutrient Agar (NA), then transferred to 10 mM MgCl_2_ and agitated for at least 2 h to disrupt biofilm and increase cell suspension homogeneity. The culture was washed twice (7000 rpm, 3 min) and adjusted to OD_600_ = 0.218. For co-infiltration, *A. tumefaciens* GV3101 (OD_600_ = 0.6) and *X. citri* (OD_600_ = 0.109) were mixed in equal volumes and infiltrated into *N. benthamiana* leaves as described above. As for disease progression assay, 15 plants per *Agrobacterium*-mediated transient expression gene were infiltrated. The HR response was scored 6–17 dpi using an HR index assigned to each of the six leaf positions and normalized per plant adapted from the DI [43].

### 4.7. In Silico Protein Structure Prediction and Subcellular Targeting

Tertiary structures of *Pt*GASA6, *Pt*GASA8, and *Pt*GASA10 were predicted using AlphaFold2 [46] implemented in ColabFold (v1.5.5) [48,49]. Models were visualized with NGL Viewer [50].

Prediction of transmembrane domains was performed using DeepTMHMM [86], and signal peptide structure was compared with *S. tuberosum* GASA orthologs (*St*SN1, *St*SN2, *St*SN3) for which subcellular localization is experimentally validated by confocal microscopy, except for *St*SN2 [18,30].

## 5. Conclusions

This study provides a comprehensive pangenomic characterization of the SNAKIN/GASA gene family in citrus rootstock species, identifying 67 novel genomic variants grouped into three conserved subfamilies. Expression profiling revealed tissue-specific patterns, with higher transcript abundance in juvenile and floral tissues of tolerant rootstocks. To assess the antimicrobial effect of selected GASA genes, it was designed an *in planta* assay based on *N. benthamiana* agroinfiltration. *Pt*GASA6, 8, and 10 showed 9.28-, 6.72-, and 5.78-fold increases in expression relative to the control, respectively. These functional assays demonstrated that transient overexpression of *Pt*GASA6 and *Pt*GASA10 in *N. benthamiana* significantly reduced disease progression caused by *P. syringae* pv. *tabaci* and enhanced hypersensitive response against *X. citri*, whereas *Pt*GASA8 showed no consistent effect. Structural predictions indicated conserved helix-turn-helix motifs and differential subcellular targeting, suggesting distinct antimicrobial mechanisms among subfamilies. *Pt*GASA6 enhanced the hypersensitive response by 30% more than *Pt*GASA10, whereas *Pt*GASA10 delayed necrosis by 40% more than *Pt*GASA6. Therefore, a reduction in disease symptoms is observed. Thus, functional evidence is provided for two members from *P. trifoliata* modulating plant defense in vivo through distinct outcomes: specifically, *Pt*GASA6 accelerated hypersensitive response (HR), whereas *Pt*GASA10 delayed disease progression in a model system. Together with the mentioned expression profiling, promoter cis-element prediction, and structural/topological inference, these results position citrus GASA genes as candidates for further studies and for potential breeding and biotechnological strategies aimed at improving broad-spectrum bacterial disease resistance in citrus. Future work should validate these genes in cultivars, explore dose-dependent responses, and assess genome editing approaches to optimize their expression and functional efficiency.

## Figures and Tables

**Table 1:** Identification and characterization of citrus GASA genes in 8 different citrus species: *Cs* (*Citrus sinensis*), *Cp*×*Pt* (*Citrus paradisi* × *Poncirus trifoliata*), *Cl* (*Citrus limon*), *Cj* (*Citrus jambhiri*), *Clia* (*Citrus limonia*), *Cw* (*Citrus warburgiana*), *Ca* (*Citrus aurantium*), and *Pt* (*Poncirus trifoliata*). CDS: coding sequence (in number of base pairs).

	Sequence Name	Gene ID	Exons	CDS	Protein (aa)
Subfamily I	*Cs*GASA6	LOC102614811	2	267	88
	*Cp*×*Pt*GASA6	OP728331	2	267	88
	*Cl*GASA6	OP728332	2	267	88
	*Cj*GASA6	OP728333	2	267	88
	*Clia*GASA6	OP728334	2	267	88
	*Ca*GASA6	OP728335	2	267	88
	*Pt*GASA6	OP728336	2	267	88
	*Cs*GASA13	LOC102619143	2	267	88
	*Cp*×*Pt*GASA13	OP791894	2	267	88
	*Cl*GASA13	OP791895	2	267	88
	*Cj*GASA13	OP791896	2	267	88
	*Clia*GASA13	OP791897	2	267	88
	*Cw*GASA13	OP791898	2	267	88
	*Ca*GASA13	OP791899	2	267	88
	*Pt*GASA13	OP791900	2	267	88
Subfamily II	*Cs*GASA8	LOC102628220	3	315	104
	*Cp*×*Pt*GASA8	OP947003	3	315	104
	*Cl*GASA8	OP947004	3	315	104
	*Cj*GASA8	OP947005	3	315	104
	*Clia*GASA8	OP947006	3	315	104
	*Cw*GASA8	OP947007	3	315	104
	*Ca*GASA8	OP947008	3	315	104
	*Pt*GASA8	OP947009	3	315	104
	*Cs*GASA9	LOC102627925	3	342	113
	*Cl*GASA9	OP946990	3	342	113
	*Pt*GASA9	OP946991	3	342	113
	*Cs*GASA9-like	LOC102628717	3	303	100
	*Cp*×*Pt*GASA9-like	OQ053283	3	303	100
	*Cl*GASA9-like	OQ053284	3	303	100
	*Cj*GASA9-like	OQ053285	3	226	73 (partial CDS)
	*Clia*GASA9-like	OQ053286	3	226	73 (partial CDS)
	*Cw*GASA9-like	OQ053287	3	303	100
	*Ca*GASA9-like	OQ053288	3	303	100
	*Pt*GASA9-like	OQ053289	3	306	101
	*Cs*GASA2	LOC102628910	4	351	116
	*Cp*×*Pt*GASA2	OP946981	2	213	65 (partial CDS)
	*Cl*GASA2	OP946982	4	213	65 (partial CDS)
	*Pt*GASA2	OP946983		351	116
	*Cs*GASA11	LOC102631482	4	504	167
	*Cp*×*Pt*GASA11	OP946975	2	408	125 (partial CDS)
	*Cl*GASA11	OP946976	2	396	130 (partial CDS)
	*Clia*GASA11	OP946977	2	396	135 (partial CDS)
	*Cw*GASA11	OP946978	2	408	135 (partial CDS)
	*Ca*GASA11	OP946979	2	396	130 (partial CDS)
	*Pt*GASA11	OP946980	4	504	167
	*Cs*GASA7	LOC102625142	4	432	143
	*Cp*×*Pt*GASA7	OP947000	4	345	114
	*Cl*GASA7	OP947001	4	345	114
	*Pt*GASA7	OP947002	4	345	114
	*Cs*GASA5	LOC102625142	3	324	113
	*Cp*×*Pt*GASA5	OP946984	3	324	113
	*Cl*GASA5	OP946985	3	327	114
	*Cj*GASA5	OP946986	3	324	113
	*Clia*GASA5	OP946987	3	324	113
	*Ca*GASA5	OP946988	3	324	113
	*Pt*GASA5	OP946989	3	324	113
Subfamily III	*Cs*GASA15	LOC102625588	3	286	95
	*Cl*GASA15	OP946995	3	285	95
	*Pt*GASA15	OP946996	3	285	95
	*Cs*GASA1	LOC102629636	4	342	113
	*Cp*×*Pt*GASA1	OQ053290	4	342	113
	*Cl*GASA1	OQ053291	4	342	113
	*Pt*GASA1	OQ053292	4	348	115
	*Cs*GASA10	LOC102618255	3	288	95
	*Cp*×*Pt*GASA10	OP830506	3	288	95
	*Cl*GASA10	OP830507	3	288	95
	*Pt*GASA10	OP830508	3	288	95
	*Cs*GASA12	LOC102611788	4	324	107
	*Cp*×*Pt*GASA12	OQ053280	4	321	106
	*Cl*GASA12	OQ053281	4	324	107
	*Pt*GASA12	OQ053282	4	321	106
	*Cs*GASA3	LOC102626694	3	312	103
	*Cp*×*Pt*GASA3	OP946997	3	312	103
	*Cl*GASA3	OP946998	3	312	103
	*Pt*GASA3	OP946999	2	312	103
	*Cs*GASA14	LOC102625321	3	351	116
	*Cp*×*Pt*GASA14	OP946992	3	286	95
	*Cl*GASA14	OP946993	3	285	95
	*Pt*GASA14	OP946994	3	285	95
	*Cs*GASA18	LOC102630614	4	357	118
	*Cp*×*Pt*GASA18	OP830509	4	357	118
	*Cl*GASA18	OP830510	4	357	118
	*Pt*GASA18	OP830511	4	357	118

## Data Availability

The original contributions presented in this study are included in the article. Further inquiries can be directed to the corresponding authors. All the data are publicly available. Germplasm accessions are available at INTA’s active germplasm banks of EEA INTA Bella Vista, Corrientes, Argentina or EEA INTA Concordia, Entre Ríos, Argentina. Additional related data not cited in this paper can be found in Bekier, Florencia PhD thesis [89].

## Supplementary Materials

The following supporting information can be downloaded at: https://www.mdpi.com/article/10.3390/plants15030425/s1: Table S1: List of BioProjects analyzed and description of their specific experimental conditions for gene expression. Table S2: Predicted cis-regulatory elements in GASA6, 8, and 10 predicted promoter sequences from *C. sinensis*, *C. limon*, and *P. trifoliata*. Table S3: Statistical differences between GASA6 and 10 dynamic responses regarding disease and HR development. Table S4: List of primers employed for the evaluation of gene expression by RT-qPCR for members of the SNAKIN/GASA gene family. Figure S1: Comparison of predicted amino acid sequences of GASA16, GASA17, and GASA18. Figure S2: Genomic distribution of GASA genes in chromosomes from *P. trifoliata*, *C. limon*, and *C. sinensis*. Figure S3: Quantification of PtGASA6 and 8 and overexpression in agroinfiltrated *N. benthamiana*. Figure S4: Statistical differences between GASA6 and 10 dynamic responses regarding disease and HR development. Refs. [39,40,41,75,87,88] are cited in Supplementary Material.

## Abbreviations

The following abbreviations are used in this manuscript:

AMP	Anti-Microbial Peptide
AUDPC	Area Under the Disease Progress Curve
AUHRPC	Area Under the Hypersensitive Response Progress Curve
DI	Disease Index
GASA	Gibberellic Acid Stimulated in Arabidopsis
HR	Hypersensitive Response
