# Supplementary material for: Pangenomic Approach for the Identification and Functional Characterization of Active GASA Antimicrobial Genes in Citrus Rootstocks for Resistance Breeding Against Bacterial Pathogens
Source: Plants (Basel). 2026 Jan 30;15(3):425. doi: 10.3390/plants15030425 (PMC12899242; doi:10.3390/plants15030425)
Supplement: Supplementary file 1 [file plants-15-00425-s001.zip › plants-4090000-supplementary.pdf]

Article

Pangenomic Approach for the Identification and Functional Characterization of Active GASA Antimicrobial Genes in Citrus Rootstocks for Resistance Breeding Against Bacterial Pathogens

Florencia Nicole Bekier [1,2,†,*], Mariana Conte [1,†], Rodrigo Machado [1,2], Lourdes Pereyra Ghidela [1,2], Natalia Inés Almasia [1], Vanesa Nahirñak [1], Nadia Frías [1], Paula del Carmen Fernández [1], Cecilia Vazquez Rovere [1], Horacio Esteban Hopp [1,2] and Gabriela Conti [1,3,*]

[1] Instituto de Agrobiotecnología y Biología Molecular (IABIMO), Unidad Ejecutora de Doble Dependencia, Consejo Nacional de investigaciones Científicas y Técnicas, Instituto Nacional de Tecnología Agropecuaria (UEDDCONICET-INTA) and Instituto de Biotecnología, Centro de Investigaciones de Ciencias Veterinarias y Agronómicas, Instituto Nacional de Tecnología Agropecuaria (INTA), Hurlingham B1686, Argentina; conte.mariana@inta.gob.ar (M.C.); rodrigomachadobirollo@gmail.com (R.M.); pereyra.lourdes@inta.gob.ar (L.P.G.); almasia.natalia@inta.gob.ar (N.I.A.); nahirnak.vanesa@inta.gob.ar (V.N.); nadia.frias.91@gmail.com (N.F.); fernandez.pc@inta.gob.ar (P.d.C.F.); vazquez.cecilia@inta.gob.ar (C.V.R.); hopp.esteban@inta.gob.ar (H.E.H.)

[2] Facultad de Ciencias Exactas y Naturales, Universidad de Buenos Aires, Intendente Güiraldes 2160, Buenos Aires C1428, Argentina

[3] Facultad de Agronomía, Cátedra de Genética, Universidad de Buenos Aires, Av. San Martín 4453, Buenos Aires C1417, Argentina

* Correspondence: bekier.florencia@inta.gob.ar (F.N.B.); conti.gabriela@inta.gob.ar or gconti@agro.uba.ar (G.C.); Tel.: +54-9-11-6424-3517 (G.C.)

† These authors contributed equally to this work.

Table S1. List of BioProjects analyzed and description of their specific experimental conditions for gene expression. **Table S2.** Predicted cis-regulatory elements in GASA6, 8 and 10 predicted promoter sequences from *C. sinensis, C. limon* and *P. trifoliata.***Table S3.** Statistical differences between GASA6 and 10 dynamic responses regarding disease and HR development. **Table S4.** List of primers employed for the evaluation of gene expression by RT-qPCR for members of the SNAKIN/GASA gene family.

Figure S1. Comparison of predicted amino acid sequences of GASA16, GASA17, and GASA18. **Figure S2.** Genomic distribution of GASA genes in chromosomes from *P. trifoliata, C. limon* and *C. sinensis.* **Figure S3.** Quantification of PtGASA6, 8 and overexpression in agroinfiltrated *N. benthamiana.* **Figure S4**. Statistical differences between GASA6 and 10 dynamic responses regarding disease and HR development.

Table S1. List of BioProjects analyzed and description of their specific experimental conditions for gene expression. All filtered BioProjects correspond to citrus plants challenged with HLB under various conditions and comparative analyses. Extracted from the DDBJ, EMBL–EBI, and NCBI databases.

BioProject	Description	Publication
PRJNA348468	Temporal analysis of 24 *C. limon* samples over time for early and late specimens	[40]
PRJNA417324	Temporal analysis of 32 leaf samples of *C. sinensis*, infected vs. non-infected (56, 126, 182, and 322 dpi)	[40]
PRJNA574168	Temporal analysis of 8 samples of Mexican lime (*Citrus aurantifolia*). Evaluation at 9- and 11-months post-infection	[87]
PRJNA629966	Temporal analysis of 23 root samples of *C. sinensis* (Kuharske Carrizo rootstock). Evaluation at 0, 3, and 9 dpi, supplemented with Hoagland after infection	[88]
PRJNA640485	Comparative analysis of mandarin supplemented with *Bacillus* vs. control mandarin plants	[41]
PRJNA645216	*C. sinensis* L. Osbeck plants probed by diseased or healthy psyllids at days 1 and 5 post-infection	[41]
PRJNA739184	Infected Valencia orange samples collected across different seasons	[39]
PRJNA739186	Infected Sugar Belle mandarin samples collected across different seasons	[39]
PRJNA755969	Comparative analysis of 6 leaf samples from infected finger lime (*Citrus australasica*) vs. Valencia (*C. sinensis*)	[39]
PRJNA780217	Orange protoplasts treated with H_2O_2 (infection simulation): comparison between GA-treated and untreated samples (8 samples)	[39]

Table S2. Predicted cis-regulatory elements in GASA6, 8 and 10 predicted promoter sequences from *C. sinensis, C. limon* and *P. trifoliata.* They were analyzed using the database of Plant Cis-Acting Regulatory DNA Elements (PlantCARE) to predict their cis-regulatory sequences [76]

		GASA6 *C.sinensis*	GASA6 *C. limon*	GASA6 *P. trifoliata*	GASA8 *C.sinensis*	GASA8 *C. limon*	GASA8 *P. trifoliata*	GASA10 *C.sinensis*	GASA10 *C. limon*	GASA10 *P. trifoliata*	
Stage	Cell cycle	0	0	0	0	0	0	0	0	1	MSA-like
	Circadian	0	0	0	0	0	0	1	1	1	circadian
Tissue	Seed	0	0	0	0	0	0	0	1	0	RY-element
	Meristem	0	0	0	0	0	0	1	1	1	CAT-box
	Palisade	0	0	0	1	1	1	0	0	0	HD-Zip 1
	Endosperm	1	1	0	0	0	0	0	1	0	GCN4_motif
Phytohormone	Zein	0	0	0	0	0	0	0	1	0	O2-site
	Salicilic acid	0	0	0	0	1	0	0	0	2	TCA-element
	Auxin	2	1	0	0	0	0	0	0	0	AuxRR-core
	Abscisic acid	4	2	2	1	0	0	1	2	0	ABRE
	Gibberellin	0	1	1	0	1	0	0	1	0	P-box
Environment	MeJA	2	0	1	1	1	1	1	0	1	CGTCA-
	Anaerobic	1	1	1	1	1	1	0	0	0	ARE
	Low-	1	0	2	0	0	0	0	1	0	LTR
	Light	0	0	0	0	0	0	0	1	0	Sp1
		0	0	0	0	0	0	0	0	1	AT1-motif
		0	0	0	3	0	0	1	0	1	TCT-motif
		0	0	0	1	0	1	0	0	0	GA-motif
		0	0	0	0	1	0	2	1	4	GT1-motif
		1	1	1	0	0	0	0	0	0	chs-CMA2a
		0	0	0	0	0	0	0	1	0	chs-CMA1a
		0	0	0	1	0	1	0	0	0	AF1 binding
		0	1	1	0	0	0	0	0	0	GTGGC-
		1	1	1	1	0	0	0	0	0	LAMP-
		1	0	1	0	0	0	0	0	0	AE-box
		5	3	2	4	2	4	5	2	5	Box 4
		4	2	2	2	1	1	1	3	0	G-box
		1	1	1	0	0	0	2	1	2	I-box
		1	1	1	1	1	1	0	0	0	ATCT-motif
		1	1	1	2	0	1	1	1	1	GATA-motif

Table S3. Statistical differences between GASA6 and 10 dynamic responses regarding disease and HR development. Ratios of *Pt*GASA6 or 10 disease and HR symptom evolution versus their respective controls were calculated from agroinfiltration experiments described in Figures 6-11. Statistical significance was determined using the non-parametric Wilcoxon rank-sum test with continuity correction and Bonferroni adjustment relative to the control with: $p < 0.05$, $p < 0.01$ and $p < 0.001$. AUDPC: area under the disease progress curve, AUHRC: area under the hypersensitive response progress curve

Gene	Disease ratio	HR ratio
*Pt*GASA6 ratio	1.1	1.82
*Pt*GASA10 ratio	1.46	1.24
*Pt*GASA6/PtGASA10	1.32727273	1.46774194

AUDPC – Wilcoxon Rank				AUHRPC – Wilcoxon Rank			
	***Pt*GASA10**	***Pt*GASA6**	***Pt*GASA8**		**Control**	***Pt*GASA10**	***Pt*GASA6**
***Pt*GASA6**	0.0027	–	–	***Pt*GASA10**	0.03265	–	–
***Pt*GASA8**	0.00013	0.00341	–	***Pt*GASA6**	0.00029	0.03948	–
**Control**	3.60E-05	0.01347	0.87082	***Pt*GASA8**	100.000	0.68871	0.00319

Table S4. List of primers employed for the evaluation of gene expression by RT-qPCR for members of the SNAKIN/GASA gene family.

Gen	Sequence (5′3′)
Housekeeping_FBOX	TTGGAAACTCTTTCGCCACT
	CAGCAACAAAATACCCGTCT
GASA1/SN8	TCTTCTCCAAAGAATAATGGCCAGA
	TATAAACTGATACCATTAATTCAAGGG
GASA6/SN1	CTCTTCTTCAGGAAATTCTGAGA
	AAGAGAATGTAATTTTAAGGACACT
GASA7/SN17	CACACTCCTATGCCTGCAGT
	ACACATCACTCGCTCCATCC
GASA8/SN2	CAGGTGCCAGTTATCGTCGA
	GTATCATGTATCATGCACAATCA
GASA9/SN3	CTGACGGGTTCGCTTCTCAT
	GCACATGCTCCGTCACAATC
GASA9_like/SN4	AGGTGCCGATTATCGTCGAG
	CGTTTCAACTTAAAATATTTCAAG
GASA10/SN9	GTAGAAGACAATACACATGGCCAC
	ATTCTCAATCAAGGGCATTTGGG
GASA11/SN10	TTCGGCTGGGTTCTGTCTTC
	GGTGGATAAGTGGTGGGTGG
GASA12/SN14	GACCTGGGACTGTCAAGAGC
	TGTTGTAGCAAGGGCACACA
GASA13/SN11	AGTCACTTACTTGAAAGGAAAGCA
	CTCCTGCTAGCTTGCACCTT

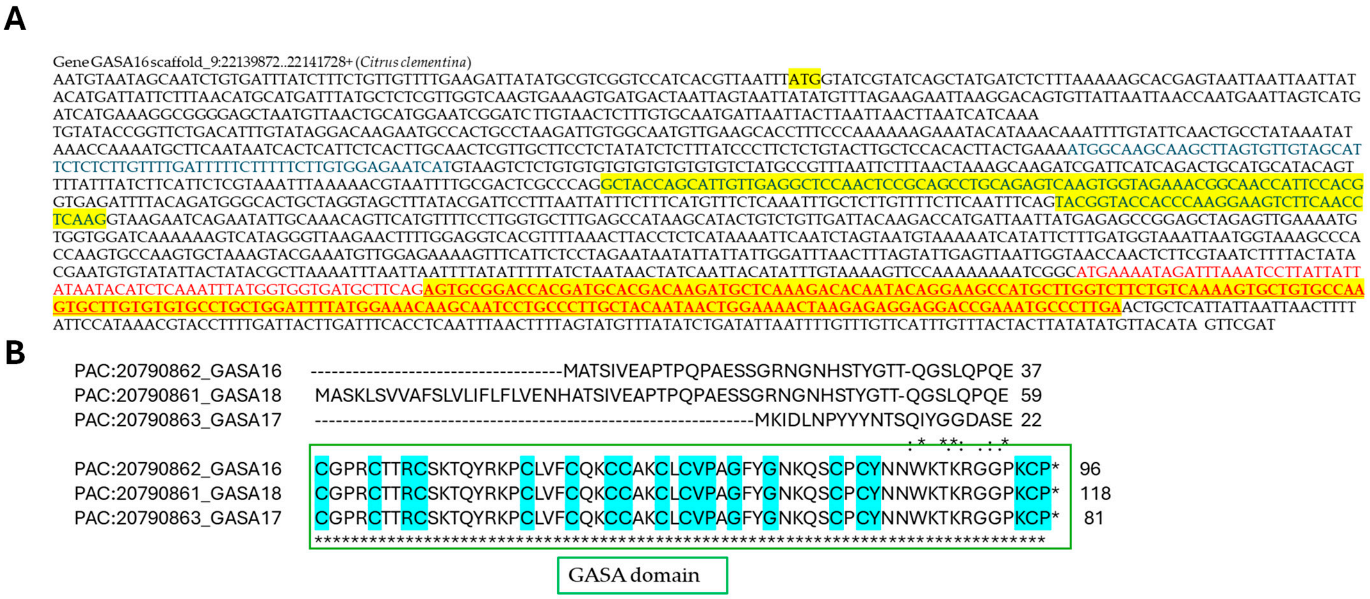

Figure S1: Comparison of predicted amino acid sequences of GASA16, GASA17, and GASA18. The three genes share the same DNA sequence in the region containing the 12 diagnostic cysteine residues and the conserved GASA domain. Differences lie mainly in the position of the ATG start codon and minor variable regions, generating alternative reading frames at the 5′ end leading to discrepancies in bioinformatic annotations as some pipelines interpret these sequences as a single gene, while others distinguish them as three separate loci. This explains why several *Citrus sinensis* BioProjects annotate only one gene in this region, whereas *Citrus clementina* projects annotate three. A similar situation may occur for GASA4 due to alternative splicing. **(A)** Comparison of the DNA sequence of GASA16 from *Citrus clementina* with GASA17 and GASA18. The three exons of GASA16 are shown on a yellow background. The single exon of GASA17 is highlighted in thin and thick red characters, while the four exons of GASA18 are shown in thick blue and red characters. Regions shared by the three genes are underlined. **(B)** Alignment of the predicted protein sequences of GASA16, GASA17, and GASA18. All proteins conserve the 12 characteristic cysteine residues, the C-terminal KCP motif, and additional highly conserved amino acids, fully matching the GASA domain.

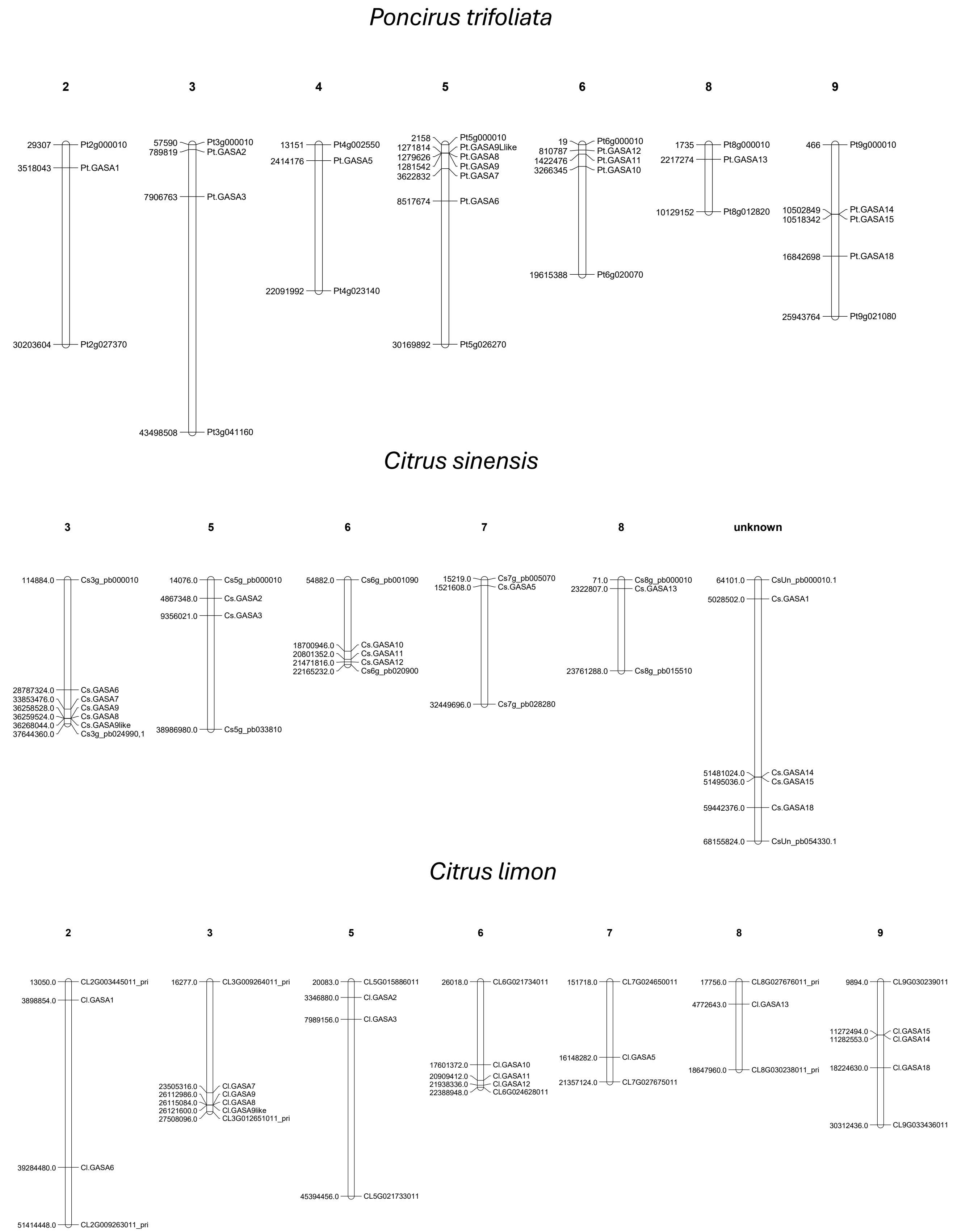

Figure S2. Genomic distribution of GASA genes in chromosomes from *P. trifoliata*, *C. limon* and *C. sinensis*. Maps were performed using MapChart [75].

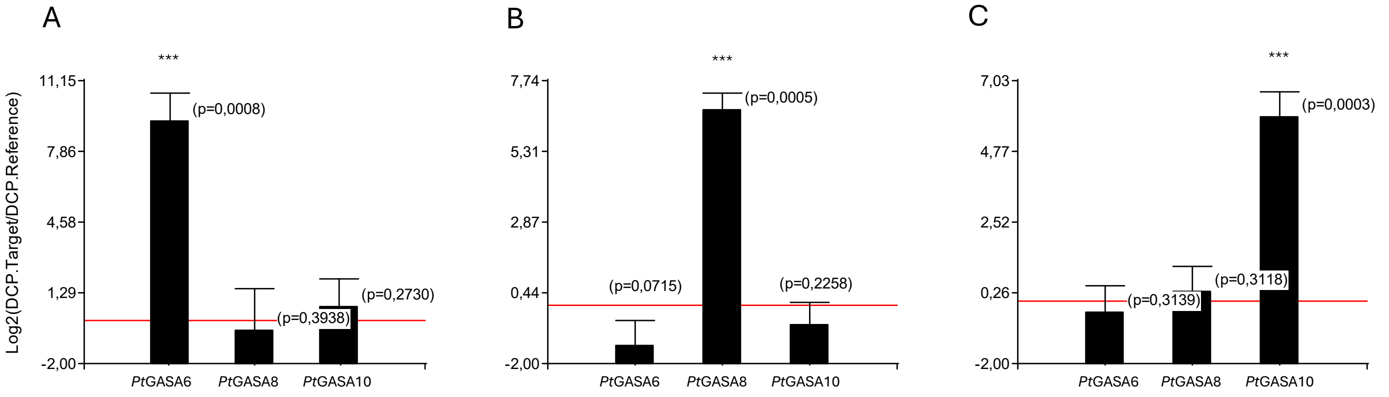

Figure S3. Quantification of *Pt*GASA6, 8 and 10 overexpression in agroinfiltrated *N. benthamiana*. Expression was quantified in leaf tissue collected 48 h after infiltration with *A. tumefaciens* GV3101 expressing *Pt*GASA6, *Pt*GASA8, or *Pt*GASA10 under the constitutive promoter 35S. Transient ectopic expression of *Pt*GASA6 **(A)**, *Pt*GASA8 **(B)**, and *Pt*GASA10 **(C)** were confirmed. As expected, overexpression of *Pt*GASA6, *Pt*GASA8, and *Pt*GASA10 was observed relative to the control for each corresponding gene. In contrast, no overexpression was detected for non-target genes. Significant differences were determined using the FgStatistics software. Statistical significance is indicated by asterisks as *** $p < 0.001$.

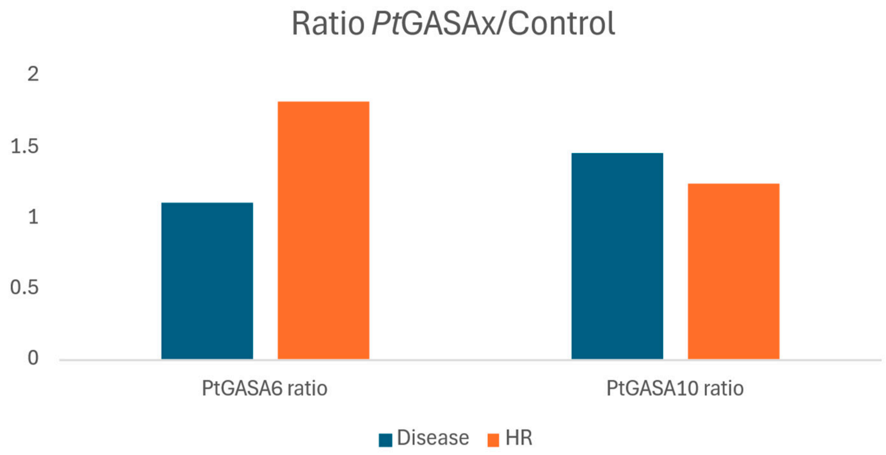

Figure S4. Statistical differences between GASA6 and 10 dynamic responses regarding disease and HR development. It corresponds to the data shown in Supplementary Table S3. Ratios of *Pt*GASA6 or 10 disease and HR symptom evolution versus their respective controls were calculated from agroinfiltration experiments described in Figures 5-10. Statistical significance was determined using the non-parametric Wilcoxon rank-sum test with continuity correction and Bonferroni adjustment relative to the control with: $p < 0.05$, $p < 0.01$ and $p < 0.001$. AUDPC: area under the disease progress curve, AUHRC: area under the hypersensitive response progress curve.

References

39. Weber, K.C.; Mahmoud, L.M.; Stanton, D.; Welker, S.; Qiu, W.; Grosser, J.W.; Levy, A.; Dutt, M. Insights into the Mechanism of Huanglongbing Tolerance in the Australian Finger Lime (Citrus Australasica). *Front Plant Sci* 2022, *13*, 1–23, doi:10.3389/fpls.2022.1019295.

40. Chin, E.L.; Ramsey, J.; Saha, S.; Mishchuk, D.; Chavez, J.; Howe, K.; Zhong, X.; Flores-Gonzalez, M.; Mitrovic, E.; Polek, M.; et al. Multi-Omics Comparison Reveals Landscape of Citrus Limon and Citrus Sinensis Response to ' Candidatus Liberibacter Asiaticus' . *PhytoFrontiersTM* 2021, *1*, 76–84, doi:10.1094/phytofr-09-20-0018-r.

41. Wei, X.; Mira, A.; Yu, Q.; Gmitter, F.G. The Mechanism of Citrus Host Defense Response Repression at Early Stages of Infection by Feeding of Diaphorina Citri Transmitting Candidatus Liberibacter Asiaticus. *Front Plant Sci* 2021, *12*, 1–22, doi:10.3389/fpls.2021.635153.

75. Voorrips, R.E. MapChart: Software for the Graphical Presentation of Linkage Maps and QTLs. *Journal of Heredity* 2002, *93*, 77–78, doi:10.1093/jhered/93.1.77.

87. Arce-Leal, Á.P.; Bautista, R.; Rodríguez-Negrete, E.A.; Manzanilla-Ramírez, M.Á.; Velázquez-Monreal, J.J.; Santos-Cervantes, M.E.; Méndez-Lozano, J.; Beuzón, C.R.; Bejarano, E.R.; Castillo, A.G.; et al. Gene Expression Profile of Mexican Lime (Citrus Aurantifolia) Trees in Response to Huanglongbing Disease Caused by Candidatus Liberibacter Asiaticus. *Microorganisms* 2020, *8*, doi:10.3390/microorganisms8040528.

88. Liu, C.; Li, T.; Cui, L.; Wang, N.; Huang, G.; Li, R. OrangeExpDB: An Integrative Gene Expression Database for Citrus Spp. *BMC Genomics* 2024, *25*, 1–7, doi:10.1186/s12864-024-104
